# Comparing Likert and visual analogue scales in ecological momentary assessment

**DOI:** 10.3758/s13428-025-02706-2

**Published:** 2025-07-02

**Authors:** Jonas M. B. Haslbeck, Alberto Jover Martínez, Anne J. Roefs, Eiko I. Fried, Lotte H. J. M. Lemmens, Esmee Groot, Peter A. Edelsbrunner

**Affiliations:** 1 Psychological Methods Group, University of Amsterdam, Amsterdam, The Netherlands; 2 Department of Clinical Psychological Science, Maastricht University, Maastricht, The Netherlands; 3 Department of Psychology, Leiden University, Leiden, The Netherlands; 4 Department of Humanities, Social and Political Sciences, ETH Zurich, Zürich, Switzerland; 5 Department of Psychology, LMU Munich, München, Germany

**Keywords:** Measurement, Response scale, Likert scale, Visual analogue scale, Ecological momentary assessment

## Abstract

Measuring subjective experiences in ecological momentary assessment (EMA) studies has become pervasive in psychological science. A design choice that has to be made in all of these studies is which response scale to use. However, to date there is little guidance on this choice in the context of EMA. As a first step towards understanding the effects of different response scales, we experimentally vary the response scale and assess whether the resulting time series of subjective experiences are systematically different. We conducted a between-person experiment comparing a seven-point Likert scale ($n=63$) with a Visual Analogue Scale (VAS; $n=56$) in an EMA study measuring affective states over 14 days. Using Bayesian multilevel models, we found that the VAS resulted in moderately higher within-person item means, lag-0 correlations, lag-1 autocorrelations, as well as lower within-person skewnesses and response frequencies of exact zeros. We found the largest difference in correlations with external criteria related to psychopathology, where correlations for the VAS were much higher. We did not observe reliable differences in within-person item variances, root mean squared successive differences, missing data, duration of measurements, and ratings about the experiences with the EMA survey. Apart from higher within-person means and higher correlations with external criteria in the VAS group, the differences were relatively small. While more research on response scales in EMA is needed, based on our results we conclude that the VAS should be preferred in studies aiming at capturing affective states relating to general psychopathology, as well as for items whose variation occurs close to scale limits. We conclude by discussing how our findings may contribute to a larger research agenda that addresses the fit of different response scales for different research aims.

## Introduction

Intensive longitudinal measurements of subjective experience collected in ecological momentary assessment (EMA) studies have become a common type of data in psychological research (Kuppens et al., 2022; Miller, 2012; Conner and Barrett, 2012; Trull and Ebner-Priemer, 2014; Hamaker and Wichers, 2017). This raises many questions about the optimal design of EMA studies (Stone et al., 2023; Gabriel et al., 2019; Wrzus and Neubauer, 2023; Hufford, 2007; Eisele et al., 2022), including acceptable burden (Wrzus and Neubauer, 2023), the time interval between measurement points (De Vries et al., 2021), the frequency of measurements (Haslbeck and Ryan, 2022), and the wording of survey items (Stone et al., 2023; Kuppens et al., 2022). Another question is which response format should be used. Currently, researchers typically use either a Likert scale or a Visual Analog scale (Haslbeck et al., 2023). Given the widespread use of these scales for EMA research, the choice of the response scale should be based on theories and empirical data about how they affect participants’ responses and the resulting data. However, to date, no study has specifically compared these two scales within the context of EMA.

There is a considerable body of research comparing different response scales in cross-sectional survey studies. Many studies took the approach of assuming a factor-model as a measurement model and focused on the reliability of measurements using multiple indicators, generally finding that increasing the number of response categories of a Likert scale to about five increases reliability (Maydeu-Olivares et al., 2009; Hilbert, 2015; Alan and Atalay Kabasakal, 2020), but that it does not increase further with more than five response categories (Simms et al., 2019; Lozano et al., 2008; Hilbert, 2015). Similarly, factor loadings on a common latent variable tend to be higher with a greater number of response categories (Xu and Leung, 2018). There are exceptions to this general finding, such as Bendig (1953), who found that reliability was similar for 2, 5, 6 and 9 response categories, but decreased with 11 categories. Criterion validity may also increase as the number of response categories increases (Taherdoost, 2019). Typically, correlations of scale scores with external criteria increase until four response categories, but remain at similar levels with more than five categories (Lozano et al., 2008; Simms et al., 2019). Overall, we conclude from studies using cross-sectional survey designs that reliability and correlations with external criteria tend to increase as the number of response categories on a Likert scale is increased up to about five categories.

Other studies on cross-sectional surveys did not assume a measurement model, but instead compared the distributional characteristics of scale scores obtained with Likert scales and VAS. Kuhlmann et al. (2017) used a within-person design (i.e., giving the same items with different response formats to the same participants) to compare a five-point Likert scale with a VAS for three personality items in an online survey. They found similar means across these response formats, and smaller standard deviations for the VAS than for the Likert scale. Voutilainen et al. (2016) compared a five-point Likert scale with a VAS in a survey on satisfaction with medical care in a within-person design and found that the VAS items were answered faster and that the means for the VAS were lower, which in this case led to reduced ceiling effects. Van Laerhoven et al. (2004) compared a five-point Likert scale with a VAS in 6 to 18-year-old children using a within-person design with items on different topics and found more extreme responses on the VAS compared to more responses in the middle categories on the Likert scale. They also found a higher percentage of missing data on the VAS, and that the children preferred the Likert scale and found it easier to use. Hasson and Arnetz (2005) compared a five-point Likert scale with a VAS and found lower medians on the VAS for positive valence items (e.g., self-rated health and energy) but higher medians for negative valence items (e.g., work exhaustion). Consistent with Voutilainen et al. (2016), but in contrast to Van Laerhoven et al. (2004), they also found more end-aversion bias on the Likert scale, meaning that the respondents tended to select the middle categories on this scale more often compared to their distributions on the VAS. Kuhlmann et al. (2016) compared a five-point Likert scale with a VAS for 55 items on the agreement with statements and found no differences in means but slightly smaller standard deviations for the VAS items. Funke and Reips (2012) compared a five-point Likert scale to a VAS in a between-person design using semantic differential items, finding no differences in means, durations, or missing data, but higher correlations between VAS responses measuring the same construct. Finally, Averbuch and Katzper (2004) compared a five-point Likert scale to a VAS in a within-person design measuring pain intensity and found comparable means across response formats. Overall, studies comparing VAS to Likert scales found inconsistent effects for means, extreme responses at floor or ceiling, missing data, durations to complete the questionnaires, and participants’ preferences.

Drawing conclusions about which response scale to use in an EMA setting based on the above results faces two issues. First, there is considerable inconsistency in existing studies on cross-sectional surveys, suggesting context or population dependence for the effects of the response scale on these scale characteristics. Second, we do not know to what extent the results from cross-sectional surveys generalize to EMA surveys. In contrast to typical survey studies, EMA studies commonly use single items as indicators of constructs (Kuppens et al., 2022) although other approaches using multi-item EMA scales exist (e.g., Kuppens et al., 2022; Trull & Ebner-Priemer, 2014; Wright et al., 2025). Results about reliability, which are commonly inferred from estimates of internal consistencies across multiple items (Maydeu-Olivares et al., 2009; Hilbert, 2015; Alan and Atalay Kabasakal, 2020), cannot be transferred to single-item measures (Song et al., 2023). In addition to the use of single-item scales (Allen et al., 2022), in EMA studies people typically answer questions several times a day over an extended period of time, rather than answering many items once. EMA therefore typically assesses states (Steyer et al., 1999) in contrast to the traits typically assessed in cross-sectional surveys. The response scale may affect states differently than measurements of more trait-like constructs typically assessed in surveys. As a result, existing work on trait reliabilities and trait correlations with external criteria provides limited guidance on the choice of the response format for state-like measures obtained in EMA.

The only work we are aware of that investigates scale effects in EMA measures is by Haslbeck et al. (2023), who re-analyzed seven open datasets and found that there was more multimodality in studies using VAS compared to studies using five- or seven-point Likert scales. However, key limitations of this work include the consideration of only a limited range of distributional characteristics and the scale comparison being conducted across different studies, which restricts the strength of conclusions due to potential unobserved confounding at the study level. While construct validity can also be assessed using a single indicator typical in EMA studies (Song et al., 2023) there are no studies to date that compare it between different response scales. Overall, there is a lack of studies comparing the Likert and VAS response scales experimentally, which enables us to avoid the confounding of scale-effects by study characteristics such as wording, assessment context, and the assessed population.

Assessing the effect of different response scales is especially important because different response processes are often plausible. For example, with a rating scale in which all response options can receive labels (i.e., a Likert scale), there is less subjectivity in interpreting the meaning of a response category. A response option that receives the label "very little" in an item asking whether a respondent currently feels sad clearly indicates the verbal meaning of the category. Yet, respondents may differ in their frames of reference. They may either compare themselves to their own typical level of sadness, or to that of people around them, leading to responses that capture different information (Hilbert et al., 2022). Response scales without labels apart from the endpoints, such as VAS, leave more room to subjective interpretation of the linguistic meaning of a response category (e.g., what a "5" on a scale from 0 to 100 means). This may reduce the comparability of responses across individuals, as they may interpret the same response option differently. However, it can provide more detailed insights into intraindividual dynamics from a subjective perspective. Another characteristic that may affect responses is the presence of a preselected initial category. If, for example, the middle category of a response scale is preselected when respondents see an item, they may diverge from this option only if they perceive strong disagreement with this default. In general, the presence of a middle category can affect responses, since this category is often selected when respondents are undecided about their response or unsure about an item’s meaning (Kulas and Stachowski, 2009). Finally, whereas many labelled response options may be overburdening, having a sufficiently large number of response options may allow participants to capture variation in their underlying states that may not be visible when only a limited number of options is available (Ouwehand et al., 2021).

In this study, we provide, to our knowledge, the first experimental evidence for the scale effects of Likert scales vs. VAS in EMA designs. We present an identical EMA survey to two groups of university students over two weeks. In a randomized allocation, one group answers all items on a seven-point Likert scales, and the other group answers all items on a VAS. In the absence of clear theories about the impact of the response scale, we explore a wide range of characteristics between the response formats, including univariate and multivariate distributional characteristics, criterion validity, participant behavior such as response times and missing data, and subjective judgements about the perceived clarity of the EMA survey. We find that while the scales lead to overall similar distributional characteristics in the measurement data, there are also reliable differences, such as higher means and with lower skewness in the VAS, higher correlations between EMA items in the VAS, and much higher correlations between average EMA items and psychopathology scales in the VAS. We discuss the relevance of our findings for choosing the best scale in practice and suggest additional research to extend the scientific basis for this decision.

**Fig. 1:**
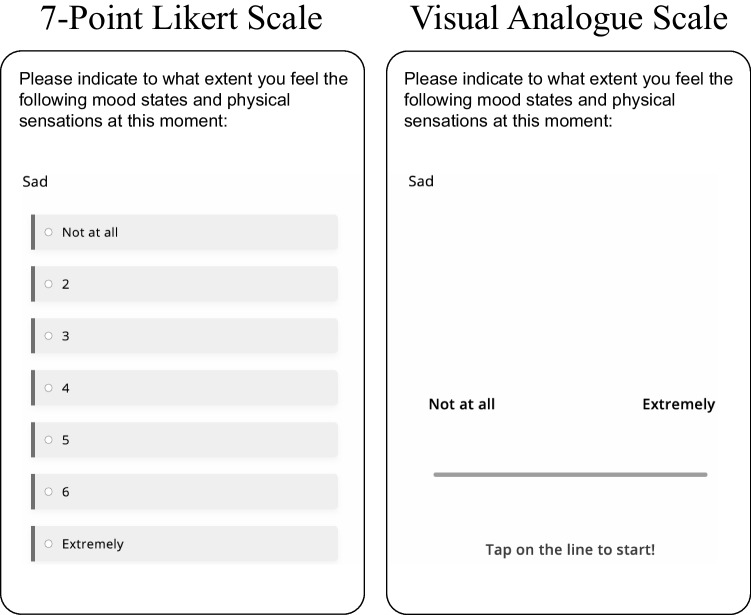
The Likert scale (*left*) and the visual analogue scale (*right*) as they appeared on the smartphones of the subjects

## Method

### Sample

We included students from various faculties at Maastricht University in the Netherlands who were at least 18 years old, owned a smartphone, and were proficient in English. In total, $N=204$ people signed up for the study, and $N=147$ completed a baseline questionnaire (see below). Of these, $N=138$ started the EMA survey and were randomly assigned to one of two groups: $n_{\text {Likert}}=73$ and $n_{\text {VAS}}=65$. Sample size is based on the number of participants that we could acquire within the time window allocated for the present study. Since this study is exploratory, we did not conduct power analysis and instead use multilevel modeling to obtain precise estimates of uncertainties that help us interpret our findings (McElreath, 2018). The study was approved by the Ethical Review Board of the Faculty of Psychology and Neuroscience of Maastricht University. The participants provided informed consent and were informed about data usage and the possibility to retract their consent for data use. The research questions and design of the project that this study was embedded in are pre-registered on AsPredicted^1^ with registration number 78277. Note that the current study’s experimental design, research questions, and analyses are not part of this preregistration.

### Measurement

Participants first completed a baseline questionnaire including the Brief Symptom Inventory (BSI), which consists of 53 items covering general psychopathology (Derogatis and Melisaratos, 1983), and the 21-item version of the Depression Anxiety Stress Scale (DASS-21; Brown et al., 1997). On the BSI, participants indicate on a five-point scale how much they have been bothered by the symptom in the last week. On the DASS, participants indicate for each of seven symptoms per sub-scale on a scale ranging from 0 = “did not apply to me” at all to 3 = “applied to me very much”, or “most of the time” to which extent each symptom applied to them during the last week. For the purposes of this study, we do not assume that these scales are generated by a single common cause and that they therefore fit a one-factor model. Instead, we assume a formative measurement model. Accordingly, we will not report measures of reliability tied to factor models, such as Cronbach’s alpha (Edelsbrunner et al., 2025). The sum scores of these baseline measures were analyzed to examine and compare the correlations of our EMA data with these external criterion measures across the Likert and VAS response scales as indicators of convergent or criterion validity. We selected psychopathology questionnaires because mood items such as those in our study are commonly used to examine research questions related to psychopathology (Thunnissen et al., 2022; Jover Martínez et al., 2025). A full description of the baseline questionnaire can be found in Jover Martínez et al. (2024).

The EMA survey of the study included a transdiagnostic psychopathology questionnaire described in Jover Martínez et al. (2023), of which we considered 14 items in the present study. We selected items that cover commonly assessed affective states (e.g., happy, satisfied, worried, sad; see Wrzus & Neubauer, 2023) on which we could expect to find variation in our mostly non-clinical sample of university students. In the EMA survey, for each item, the participants were asked: “Please indicate to what extent you feel the following mood states and physical sensations at this moment:” (see Fig. 1). Specifically, we assess the following 14 items: Happy, Energetic, I look forward to the activities that I planned for later, I am satisfied with myself, I am satisfied with my body, Sad, Guilty, Ashamed, Disgusted, Anxious, Irritated, Physical discomfort (such as pain), Lonely, and Stressed.

The Likert group answered all items on a seven-point Likert scale, the VAS group on a 0 to 100 scale. Endpoints in both groups were labeled as “Not at all” and “Extremely”. Due to software restrictions, the Likert scale was presented vertically, as shown in Fig. 1. The VAS display had no ticks, and only when a participant touched the screen did a dot appear. Above the dot, an integer between 0 and 100 indicated the currently selected number.

For a subset of 53 (28 Likert) of the participants, we also asked six questions reflecting on their experience with the EMA survey. The questions were (1) about how difficult it was to complete all EMA surveys, (2) how burdensome the survey was, (3) how the participants judged the frequency of assessments, (4) how clear the questions were, (5) how difficult it was to know the answers to the questions, and (6) how much participation in the EMA survey affected the participant’s life. Each of the six items was answered on a seven-point Likert scale. The exact wording of these six questions and their respective response categories can be found in Appendix A.

### Procedure

We report all measures and conditions used for this study, data exclusions, and how we determined the sample sizes. Participants were recruited through various platforms such as social media (Facebook and Instagram), flyers, lectures, and the university recruitment system (SONA). The reward for participation consisted of up to €40 in vouchers, or up to €20 in vouchers and up to two research credits, depending on compliance.

Participants first completed an online screening, which checked the above-mentioned inclusion criteria and asked participants to give informed consent. After completing the online screening, participants received an email indicating their group and a participant manual explaining the study and the questionnaire items. Participants were only informed about their assigned group, but not about the aims of the study. The day after completing the online screening, participants received the baseline questionnaire and the entrance survey, in which they indicated the group they received by e-mail. Three days after completing the online screening, participants began the 14-day EMA period.

During this 14-day period, surveys were triggered six times per day. There was a 2-h interval between the surveys and the first survey was triggered between 8:00 am and 10:00 am, 9:30 am and 11:30 am, or 11:00 am and 1:00 pm, depending on the participants’ usual wake-up time as stated in the baseline questionnaire. Therefore, the participants could provide up to 84 measurement points for each item ($6 \times 14$). Surveys were triggered semi-randomly within 2-h intervals following a normal distribution (i.e., the chances of a survey being triggered in the middle of the time interval were maximized). All surveys contained the same number of items, except for the first and last surveys of the day, which contained additional items about sleep and experiences throughout the day that we do not analyze in this paper. The first and last surveys expired after 45 min, and the others expired after 20 min. Push notifications were sent when a survey was triggered, and 12 min before the survey expired. An additional push notification was sent for the first and last surveys of each day 30 min prior to expiration. The EMA study was conducted using Avicenna software (https://avicennaresearch.com/; formerly EthicaData; https://ethicadata.com/).

### Data analysis

#### Considered data characteristics

We assess differences between the Likert scale and the VAS on four sets of characteristics of the EMA time series.

First, we compare univariate characteristics of the EMA items between the two response scales. Typical univariate characteristics include the mean, variance, and skewness. Since the study by Haslbeck et al. (2023) suggested increased multimodality in EMA studies using VAS compared to Likert scales, we also examine multimodality. The univariate characteristics provide an overall impression of how the different response scales affect the item distributions, which may help to develop first hypotheses about how the response scales affect the response process.

Second, further univariate characteristics that are of special relevance in EMA studies are within-person indicators of stability and variation over time. Specifically, since the temporal dynamics are often the focus of EMA studies (e.g., Mansueto et al., 2022; Ryan et al., 2023), we investigate how the root mean squared successive differences (RMSSDs; capturing how much responses to an item change from one time point to the next) and the lag-1 autocorrelations (capturing how much items are correlated from one time point to the next) differ across the two scales. In addition, initial elevation bias, which is the phenomenon that responses in intensive longitudinal data are elevated during the first few assessments, has received recent interest in the field of EMA research (Anvari et al., 2023) and we will compare its occurrence across response scales.

Third, analogous to modeling the correlational structure between latent constructs in cross-sectional survey studies, we compare the strength and structure of the correlations between the different terms in our EMA data. EMA research focuses on within-person rather than between-person correlational structures. Accordingly, we compare the average within-person correlations of each item with all other items, as well as the full within-person correlation matrices including all items between the two response scales. In research using multi-item scales, the correlational structure between latent constructs allows examining convergent and divergent validity. Similar to this approach, in examining the within-person correlational structure between the single-item measures in our study, we examine whether inter-item correlations align with our expectations (i.e., positive correlations among positive as well as among negative valence items, and negative correlations between positive and negative valence items), and we compare these patterns across response scales.

Fourth, as another important indicator for validity, we will compare the items’ correlations with external criterion variables across the two response scales. Specifically, we correlate the within-person means of the momentary emotion variables with the external criterion measures assessed at baseline, which are the BSI capturing general psychopathology, and the three subscales of the DASS, measuring depression, anxiety, and stress. It is not the aim of our study is to conduct a psychometric validation study of the response scales according to a specific psychometric or substantive theory. Yet, if either scale shows larger correlations with these external criteria, then this would indicate that the respective scale manages to capture more signal that relates to general psychopathology, a topic that is commonly in focus when using EMA measures of affective states (Thunnissen et al., 2022). Importantly, we do not expect that the EMA emotion means measure the same as the external criteria, nor do we expect particularly high correlations (see also Augustine & Larsen, 2012; Newman et al., 2021). Still, it only makes sense to use an external criterion that is in some meaningful way related to the measured EMA items. We think that emotion dynamics are part of the larger system giving rise to the mental health problems captured by the four external criteria scales. This type of reasoning invoking dynamical/complex systems and associated systems-level, or emergent, behaviors is often used to motivate EMA research (e.g., Borsboom et al., 2022; Cramer et al., 2016; Cui et al., 2023; Dablander et al., 2023; Helmich et al., 2024; Olthof et al., 2023; Wichers et al., 2015, 2016). We consider it plausible that the within-person means capture a relevant aspect of emotion dynamics and are therefore related to our external criteria. We do not make any assumptions about how large those correlations are, we only argue that a measurement instrument that leads to a higher correlation likely measures the emotion at hand more precisely. This is also because an alternative explanation would require a common cause affecting both the external criteria and a measurement “error” specific to that measurement instrument, a scenario we find difficult to find examples of.

Finally, in addition to these psychometric characteristics, we assess the extent to which there are scale differences in the proportion of missing measurement points and in the duration of measurements. We also analyze answers to six questions that examine participants’ experiences with the EMA survey, and compare scores across the two response scales.

#### Scale normalization

To be able to meaningfully compare Likert and VAS, we need to map them to the same scale. We do this by normalizing both scales to the interval [0, 1] before calculating any of the above measures. That is, we subtract 1 and divide by 6 in the case of the 1–7 Likert scale, and divide by 100 in the case of the VAS. Mapping both response scales to the normalized [0, 1] scale implies that we assume a ratio scale for both measurement scales. While this assumption is not always necessary to model and interpret the responses from either of the two scales separately, we require this assumption to meaningfully compare the two scales. In addition, this assumption is made by the vast majority of models typically used to model both VAS and Likert scale data, which assume that the data are continuous.

#### Data inclusion

In order to estimate the above within-person characteristics with sufficient precision, we only include participants in the analysis who provided at least 20 of the 84 possible measurements. While any cutoff is arbitrary to some extent, we choose 20 because it provides a good trade-off between including as many participants as possible and obtaining sufficiently precise estimates based on the rough ranges we would expect for the population parameters and the $\frac{1}{\sqrt{n}}$-scaling of the central limit theorem. For example, the means of the normalized scales vary between 0 and 1. Our results show that standard deviations are around 0.15. Thus, with $N_t = 20$ we would get a standard error of $\frac{0.15}{\sqrt{20}} \approx 0.035$ which we deem acceptable on a [0, 1] scale.

We applied the same criterion to measures computed over time lags (RMSSD and AR). However, for these measures, the sample sizes will be different because, if a time point is missing, it means that (1) it cannot be predicted and (2) it cannot be a predictor for the next time point. With this we ensure that all measures are based on at least 20 measurements per person.

To check data validity, we visually inspected the multivariate time series of each participant to detect any anomalies that would indicate that a participant did not adequately participate in the survey. We also investigated the within-person correlation matrices of each participant to examine whether the typical pattern of items with similar affect valence (i.e., positive or negative) correlating positively with each other but those with different affect valence negatively would be found in each person.

#### Descriptive and inferential statistics

To compare the data between the two response scales, we used descriptive statistics, as well as statistical models to estimate parameters and perform inferences about scale differences. For descriptive statistics, we computed the respective statistics (e.g., means, skewnesses, RMSSD, proportions of missing data) over time for each item for each participant.

*Bayesian multi-level modeling.* To perform statistical inferences about scale differences in the above-mentioned characteristics, we needed models that allowed us to estimate statistical parameters and compare them across the two response scales. Since for most of the modeled statistics, measurements were nested in a cross-classified manner within participants and items, we modeled them using a multi-level approach. Specifically, we used the Bayesian multilevel approach as implemented in the R-package *brms* (Bürkner, 2017). We chose this approach because it allowed us to implement a wide range of distributions that we needed to adequately model the different distributions of the characteristics considered. For example, some characteristics were bounded by definition, such as the autocorrelations and correlations $[-1, 1]$ or the standard deviation $[0, \infty ]$, or by design, such as the mean, because both the Likert scale and the VAS are bounded. Some of the characteristics represented (binomial) proportions (multimodality and missing data) or had zeros and ones in addition to the remaining part of the distribution (requiring zero- or one-inflation). We did not incorporate parameters to incorporate autoregression in the models, as this is only a concern in models parameterizing processes over time (e.g., cross-lagged models or models of within-person change; see Brossart et al., 2006; Schwartz & Stone, 1998), which we do not model here.

In addition to the large number of distribution functions, Bayesian estimation allows us to derive in a relatively straightforward way the full posterior distributions of the estimated marginal means, variances, and zero- (i.e., responses at the lower bound) as well as one- (i.e., responses at the upper bound) proportions and differences therein between the two response scales that we use to convey our results. We report these estimated marginal means instead of model parameters in many places because the former are easier to interpret, especially for less technically inclined readers.

*Specification of (random) intercepts and slopes.* In all Bayesian regression models, we included a fixed effect of the response format (Likert vs. VAS, with Likert as baseline condition representing the intercept). In addition, in all models including statistics across the different items, we included a fixed effect of item valence (negative vs. positive balance, with effects coding such that the intercept represents the average across both valence types), and its interaction with the fixed effect of the response format. Because the focus of this research is on the response scale effects, we report item valence effects only when they interacted with the response scale. In addition, in all models where this was appropriate given the design, we included random intercepts across participants and items, as well as a random slope of the response format across items and a random slope of item valence across participants, to ensure estimating the full appropriate random effects structure, to avoid bias from unmodelled effects, and improve generalizability of our results across participants and items (Barr et al., 2013; Yarkoni, 2022).

*Specification of response distributions.* To match the distributions of the different statistical indices, we implemented Gaussian error distributions for skewnesses, autocorrelations, and the initial elevation bias, a beta distribution for mean item correlations (Ferrari and Cribari-Neto, 2004), zero-inflated beta distributions (Liu and Eugenio, 2018) for means, standard deviations, and RMSSDs, zero-/one-inflated beta (Liu and Eugenio, 2018) for proportions of missing data, a Bernoulli distribution for the probability of multimodality, a lognormal distribution (Ranger et al., 2020) for response durations, and cumulative ordinal models (Bürkner and Vuorre, 2019) for the post-survey questionnaire. With these distributions, we achieved an appropriate fit, as indicated by posterior predictive model checks (see Appendix B). In the beta regression models, zero- or one inflation is required to model any zero- or one-responses, as these minimum and maximum responses are not captured by the regular beta regression parameters (Liu and Eugenio, 2018). An interpretative advantage of this is that the parameters indicating the zero- or one-inflations can be interpreted directly as the estimated proportion of zero- or one-values after back-transformation from the logit scale. We implemented all models as full distributional models (Umlauf et al., 2018), meaning that the fixed effects were specified not only for the distributions’ expectation parameters (e.g., *mu* and threshold parameters in the Gaussian, lognormal, Bernoulli, and ordinal models), but also for their variances (Gaussian & lognormal models)/precisions or shape parameters (beta regression), and for zero- or one-inflations, where appropriate. In this way, we could model effects of response format and valence not only on the means of the distributions, but also on the variation across participants and items, as well as on the amount of zero- and one-values in the respective models (Klein et al., 2015; Umlauf et al., 2018).

*Modeling relationship between EMA items and baseline scales.* To model the effects of the response scale on the correlations with the external criterion variables (BSI, DASS scales), we estimated Bayesian multivariate Gaussian regression models in which the dependent variables were the participants’ *z*-scores on the different emotions on the respective response scale and the independent variable were the *z*-scores on the respective external criterion variable. Through the *z*-standardization, we could estimate correlations between the scales and emotions and compare these between the two response scales by deriving parameters for the average absolute correlations across emotions on the respective scales.

*Software and implementation.* The models were implemented in the *brms*-package in R (Bürkner, 2017) using three Hamiltonian Monte Carlo chains with 1000 warm-up samples and between 1000 and 3000 post-warm-up samples each. Convergence was indicated by smooth posteriors, mixing in trace plots, and $\hat{R}$-values below 1.02 for all parameters. Effective sample sizes for bulks and tails were above 500 for all parameters. We used moderately informative priors for most fixed and some random effects to support model convergence and avoid estimation issues by providing priors with highest density regions that covered realistic values for the different model parameters. For all other parameters, we used the default priors in *brms*, since these appeared appropriate as weakly informative priors that do not visibly affect the posterior characteristics but support convergence. Details for all specified priors can be found in the online supplementary materials at https://github.com/jmbh/LikertvsVASPaper. As parameter estimates, we report the posterior medians with 90% highest density intervals. For statistical inference, we exclude the possibility that a parameter equals zero in the population if its 90% HDI excludes zero, and if the HDI includes zero, we interpret the estimate’s magnitude and its 90% HDI qualitatively, but do not exclude the possibility that the population parameter equals zero to avoid misinterpretations (Edelsbrunner and Thurn, 2023). If the parameter estimates indicate a notable difference, but their 90% HDIs include zero, we indicate the descriptive result to require replication in future research. We chose 90% credible intervals, because they provide 90% certainty about the parameters’ most likely locations. We considered this to be an appropriate level of certainty, particularly since this study is exploratory and we aimed at generating hypotheses for future research and thus to prevent higher beta-error (i.e., type II error) rates.

*Modeling differences in mean within-person item-correlations.* To compare mean within-person item correlations between response scales, we used a non-parametric Frequentist approach, because the Bayesian multilevel models would be too complex to implement in this setting. Specifically, we used a permutation test with $N_\text {P}=500$ permutations to compute empirical *p* values. We were not able to use a standard SEM approach in which we test an equality constraint on the covariance matrices between two groups, because this approach does not fit our nested data structure. Such an approach would require single samples from individuals, while here we have a covariance matrix for each individual.

## Results

### Final sample

After applying our data inclusion criteria, we obtained our final sample with $n_{\text {Likert}}=63$ participants and $n_{\text {VAS}}=56$ participants. For descriptive statistics and frequency distributions of demographic variables by group, refer to Appendix H. For descriptive statistics on the variables of interest comparing responders and non-responders, see Appendix I. One person in the VAS group was excluded because visual inspection showed that this person gave identical responses to all items (positive and negative valence) at each time point, which is clearly careless responding. The additional inspection of individual participants’ within-person correlation matrices showed that most participants followed the expected patterns, but there were a few participants visibly deviating from these patterns. Specifically, a few participants showed moderate positive correlations between items of different affect valence (e.g., positive correlations of “discomfort” with “looking forward”). However, we could not tell whether these patterns were unrealistic, pointing towards validity issues, or whether they represented actual variation in the affect dynamics of different persons. We therefore did not exclude these persons from the analyses. The respective correlation matrices for all participants are provided in the supplementary materials. The remaining exclusions were due to the sample size cutoff of $n=20$. The mean age of the final sample was 21.73 (SD = 3.94), and 100 identified as female. Most participants were from European countries such as the Netherlands ($n=29$) or Germany ($n=27$), but participants from other non-European countries, such as China ($n=10$), the United States of America ($n=4$), or Kazakhstan ($n=2$) also participated in the study. $n=25$ participants had been diagnosed with a mental disorder at some point in their lives. Of those diagnosed with a mental disorder, $n=11$ were taking medication during the study. The analyses of RMSSD and lag-1 autocorrelations were based on $n_{\text {Likert}}=56$ and $n_{\text {VAS}}=51$.

**Fig. 2:**
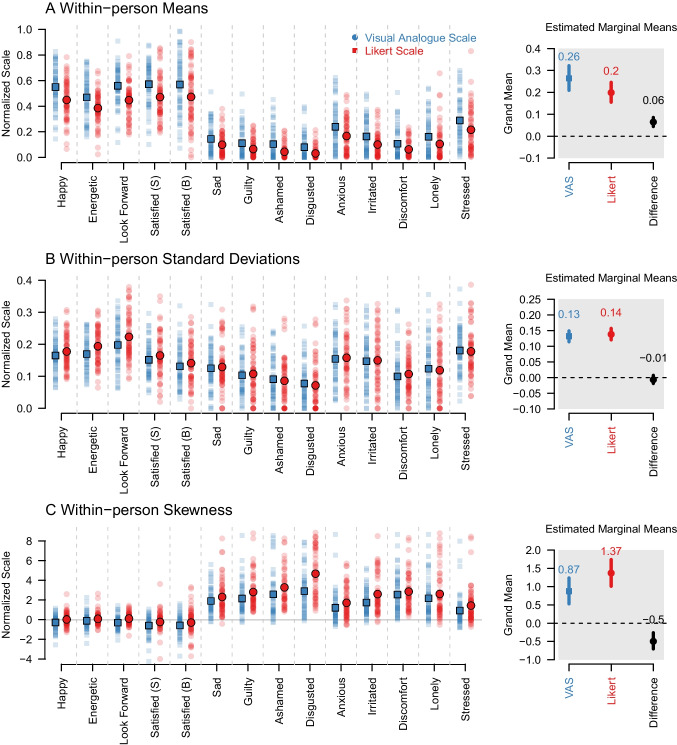
Results for **A** the within-person means, **B** the within-person SDs, and **C** the within-person skewness, for the group using a Likert scale (*red*), the VAS (*blue*). *Solid squares*/*points* are means. The *lighter points* are the raw data. The right side of each panel shows the estimated marginal means and 90% highest density intervals of the posteriors of fixed effects in the Bayesian multilevel models. Raw parameter estimates are provided in the Appendix

### Univariate distributional characteristics

#### Within-person means

Looking at the distributions of the within-person means in panel A of Fig. 2, we see that the participants in both groups (i.e., Likert and VAS) score relatively high on the positive items such as Happy and Energetic, but relatively low on the negative items such as Sad and Guilty. This is what we would expect in a relatively healthy sample, and we would expect both scales to show this qualitative pattern. Comparing VAS (blue) and Likert (red), we see that the means are higher in the VAS group for all items. This effect is strongest for the positive valence items: Happy, Energetic, Looking forward, Satisfaction with Oneself, and Satisfaction with one’s Body.

We perform inference on scale differences in within-person means using a Bayesian multilevel zero-inflated beta regression with valence, scale, and the interaction between valence and scale as fixed-effects predictors on the item level. Posteriors for all estimated fixed effects are shown in Fig. 8 in Appendix B. On the right side of panel A of Fig. 2, we summarize the posteriors of the estimated marginal means for the Likert group, the VAS group, and the difference between them. The square/point indicates the median of the posterior, while the horizontal lines indicate the 90% highest density interval (HDI). We found that the median of the posterior of the VAS group was equal to 0.26, while the median for the Likert group was equal to 0.20 and therefore, on average across items, the means were $30\%$ larger in the VAS group. Inspecting the HDI of the difference between the scales shows that it does not overlap with zero, from which we conclude that the means on the items are higher in the VAS group in the population. The regression model also included a strong main effect of valence, reflecting the clear pattern in the data of higher means for positive valence items (see Fig. 6 Appendix B). Next to the means, we also examined the proportion of exact zero values and the variance of within-person means. We found that the variance was 31% larger in the VAS group (see Appendix C) and that there were 28% fewer means of exactly zero in the VAS group (see Fig. 6 in Appendix B).

#### Within-person standard deviations

The distributions of within-person standard deviations were similar for both scales (panel B in Fig. 2). We modeled standard deviations using a Bayesian multilevel linear regression model with fixed effects for valence, scale, and the interaction between valence and scale. The posteriors of all fixed effects can be found in Appendix B. The right part of panel B shows the estimated marginal mean standard deviation for the Likert scale (0.14) and the VAS (0.13). The HDI of the difference overlapped with zero, so we cannot conclude that the standard deviations are different across scales. While we conclude that there is no visible scale effect, we found that standard deviations are higher for items with positive valence (see Fig. 8 in Appendix B).

There were no reliable scale effects for the variance across people in within-person standard deviations (see Appendix C) and there was a $27\%$ higher proportion of zeros for the Likert scale, indicating that a larger number of participants in the Likert group show no variance at all (see Fig. 8 in Appendix B). Note that this increased number of zeros must occur because it also occurred for the means.

#### Within-person skewness

For skewness, panel C of Fig. 2 shows that positive valence items have negative skew (right-skewed) and negative valence items have a much stronger positive skew (left-skewed). This is a well-known phenomenon that is at least partially due to higher means for positive items in the general population combined with the fact that both scales are bounded (see also Haslbeck et al., 2023).

The raw skewness was also somewhat higher for the Likert scale in most items (Fig. 2). We model scale differences with a Bayesian multilevel regression model, with fixed effects for valence, scale and their interaction. Posteriors for all fixed effects are reported in Fig. 14 in Appendix B. The estimated marginal means in Fig. 2 show that the skewness was $57\%$ higher for the Likert scale (1.37) than for the VAS (0.87). The HDI of the difference does not overlap with zero, from which we conclude that the raw skewness is higher for the Likert scale. We also found that raw skewness was larger for items with positive valence (see Fig. 14). We also looked at the variance of skewness and found that it was 33% smaller in the VAS group (see Appendix C).

#### Multimodality

To assess the number of modes in the item distributions, we used the same density estimation-based approach to estimate the number of modes as in Haslbeck et al. (2023). However, since the time series available in this study is only about half as long as in their paper, we slightly adapted the mode estimation algorithm by adding less noise to the data than in the original algorithm before performing the density-based modality detection. This change creates more sensitivity for detecting multimodality, but is the same for both scales, and therefore cannot corrupt the comparison. We found that $4.88\%$ of distributions were bimodal for the Likert scale, whereas $7.53\%$ were detected as bimodal for the VAS. The median of the model-implied difference is equal to $1.3\%$ and the associated HDI $[-0.00, 0.03]$ overlaps with zero, indicating a descriptive difference only, which requires replication in future research (for details see Fig. 13 in Appendix B).

**Fig. 3:**
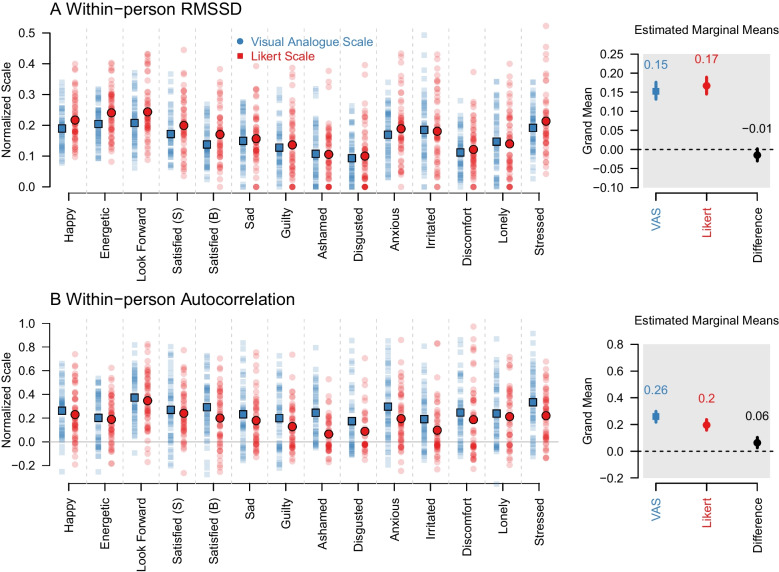
Results for **A** the within-person Root Mean Successive Squared Difference (RMSSD) and **B** the within-person autocorrelation, separately for the group using a Likert scale (*red*), the VAS (*blue*). *Solid squares/points* indicate medians, the *bars* indicate the 5% and 95% quantiles of the data. The *lighter points* are the raw data. The right side of each panel shows the estimated marginal means and 90% highest density intervals of the posteriors of fixed effects in the Bayesian multilevel models. Raw parameter estimates are provided in the Appendix

### Temporal dynamics

#### Within-person RMSSD

The RMSSDs are very similar across valence and scales (panel A in Fig. 3). We performed inference on scale differences using a Bayesian multilevel zero-inflated beta regression with fixed effects of valence, scale, and their interaction. Their posteriors are displayed in Fig. 11 in Appendix B. Deriving estimated marginal means, we see that the RMSSDs were similar across the Likert scale (0.55) and the VAS (0.56), and that the HDI of the difference overlaps with zero (HDI $[-0.02, 0.00]$), from which we conclude that the parameter estimates of the RMSSDs are very similar across the two scales, but there is a slightly higher estimate for the Likert scale that requires replication in future research. In addition, the RMSSD was higher for items with positive valence (see Fig. 11) and we found slightly ($6\%$) lower estimated marginal variance on the VAS with the HDI just excluding zero.

#### Within-person lag-1 autocorrelations

panel B in Fig. 3 shows higher within-person autocorrelations for the VAS for most items. We modeled scale differences with a Bayesian multilevel beta regression, with fixed effects of valence, scale and their interaction. The corresponding posteriors are displayed in Fig. 13 Appendix B. On the right side of panel B we show the estimated marginal means of the lag-1 autocorrelations, which are slightly lower for the Likert scale (0.20) than for the VAS (0.26). The HDI of the difference does not overlap with zero (HDI [0.02, 0.1], from which we conclude that the lag-1 autocorrelation is about $30\%$ larger for the VAS. We also found that the lag-1 autocorrelation was higher for items with positive valence (see Fig. 13). There were no reliable scale differences in the variances of the within-person autocorrelations (see Appendix C).

#### Initial elevation bias

We did not observe any initial elevation bias and therefore we also did not observe any scale differences (see Fig. 20 in Appendix E). This is also confirmed by the fact that the posteriors of the intercept and the VAS effect are extremely close to zero with little uncertainty in the corresponding Bayesian multilevel model. There were no reliable scale differences in the variances in Initial Elevation Bias (for details see Fig. 7 see Appendix C).

**Fig. 4:**
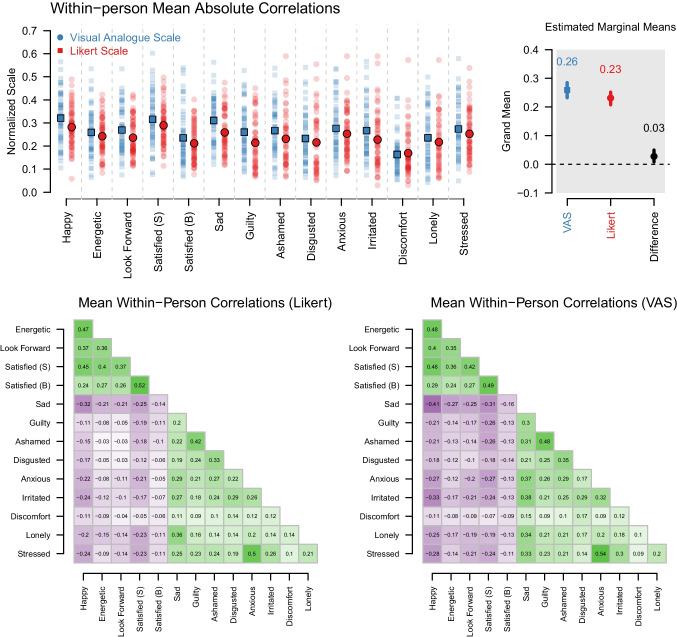
*Top row*: Within-person mean absolute correlations for each item. *Light points* are the data, *dots*/*squares* are the medians, *bars* are 95% quantiles of the data. The right side of the panel shows the estimated marginal means and 90% highest density intervals of the posteriors of fixed effects in the Bayesian multilevel models. Raw parameter estimates are provided in the Appendix.* Bottom row*: The mean within-person correlations between all variable pairs, separately for the Likert (*left*) and the VAS (*right*) group

**Fig. 5:**
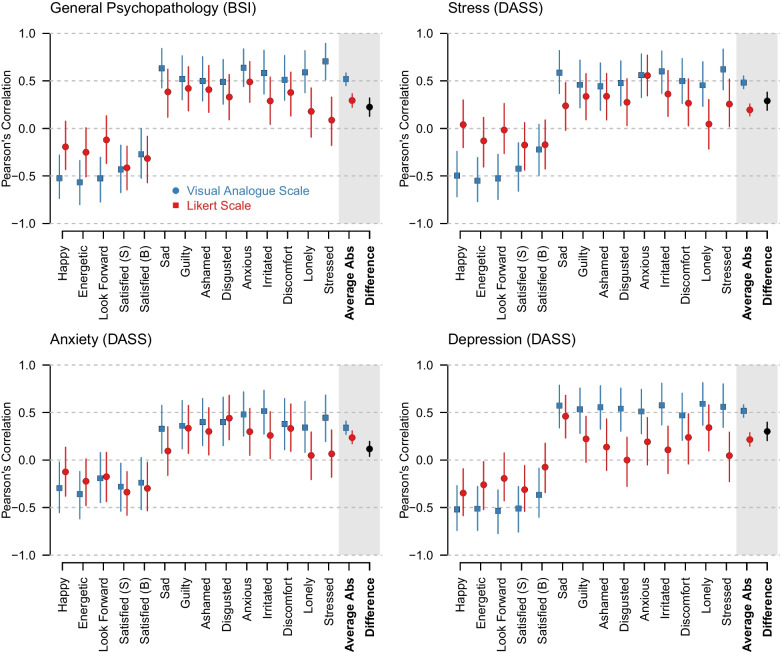
The correlation between the means of the respective ESM items, separately for the Likert scale (*red*) and the VAS (*blue*). “*Average*” indicates the effect averaged over the *absolute* correlation for all 14 emotions. “*Difference*” shows the difference between the average absolute correlations between the two scales (VAS minus Likert)

### Item correlation structure

#### Within-person mean absolute correlations

We first considered the mean absolute within-person correlation of each variable with all other variables in the top row of Fig. 4. We see that they were consistently a bit higher for the VAS. We modeled the mean absolute within-person correlation with a Bayesian multilevel beta regression with fixed effects for valence, scale and their interaction. The posteriors of all fixed effects are shown in Appendix B. In the right part of the top row we show the estimated marginal means for the mean absolute correlations for the Likert scale (0.23) and the VAS (0.26). Since the HDI of the difference does not overlap with zero, we conclude that the mean absolute correlation is about $13\%$ larger in the VAS in the population. In addition, the variance in mean absolute correlations was $8\%$ higher in the VAS condition (see Appendix C).

#### Within-person bivariate item correlations

The bottom part of Fig. 4 shows the correlations between all item pairs, averaged across participants, separately for the two measurement scales (left: Likert; right: VAS). We see that for both scales, the correlation structure showed the expected block structure, where variables with the same valence are positively correlated, while variables with different valence are negatively correlated for both response scales. We see that the absolute correlations were overall a bit higher in the VAS group, consistent with the aggregate scale effect assessed in the multilevel model. We explicitly display the differences between correlations and perform a permutation test testing the null hypothesis that the correlations are the same across scale in Fig. 21 in Appendix F. We performed a test for each of the $\frac{14(14-1)}{2}=91$ correlations and found that 22 correlations were significant with a threshold of $\alpha =0.10$, all of which had higher absolute values in the VAS group. Based on the highly consistent patterns of observed differences, we would expect that almost all correlations to be higher in the VAS group in the population, but many differences were too small to be detected with our test.

While the absolute value of correlations was larger in the VAS group, the overall structure in the two correlation matrices was very similar. We see high similarity across scales within each block, for example, the variable pairs (Happy, Energetic), (Happy, Satisfied (S)), (Satisfied (B), Satisfied (S)), and (Anxious, Stressed) showing the strongest correlations in both scales. The correlation across scales between the 91 item-wise correlations is 0.99, indicating that the correlation structure is highly similar across the two scales. We provide a more detailed comparison of the structure of the correlation matrices by displaying the relationship between correlations in the Likert and VAS groups and comparing their eigenvalue distribution in Fig. 22 in Appendix F. Consistent with the above, these results suggest that beyond the larger absolute correlations in the VAS group, the correlation structures are highly similar across the two scales.

### Criterion correlations

We perform inference on scale differences in correlations within-person means of EMA items and the sum scores of the BSI and the three DASS subscales using the multivariate Bayesian regression model with *z*-standardized independent and dependent variables described in the Methods section. Figure 5 displays the medians and HDIs of the model-implied posteriors for each correlation, separately for the two response scales.

Across response scales, we see that EMA items with positive valence correlate negatively with the constructs, while EMA items with negative valence correlate positively with the constructs. This makes sense, because each of the constructs captures symptoms of mental disorders or similar problems. We also see that the absolute correlations were consistently higher for the VAS than for the Likert scale. While the $90\%$ HDIs for most items overlapped, the pattern was very consistent across items, suggesting that the absolute correlations in the population are higher for the VAS. We quantify the aggregate effect on the right of each panel, where we show the model-estimated average absolute correlation across items with the associated $90\%$ HDI for each scale. These model estimates showed that the average absolute correlation is considerably larger for VAS in case of the BSI ($77\%$), and the Stress ($147\%$), Anxiety ($44\%$), and Depression ($140\%$) subscales of the DASS. We also display the median and HDI of the difference in this average absolute correlation (VAS minus Likert), showing large differences with all of the HDIs of the differences excluding zero. We therefore conclude that in the population the VAS scale leads to higher absolute correlations than the Likert scale. We also inspected the scatter plots behind each correlation to check for any irregularities and did not find any. We provide those in our reproducibility archive.

### Accounting for scale differences with simple thresholding process

Our results show that the two scales lead to measurements that are on several characteristics systematically different from each other. To explain such differences, one would need to develop a model for the response process triggered by each of the response scales and to study their differences. These response processes may be nontrivial and vary across people, and we mention a number of research lines to study them in the discussion section. Here, we explore to what extent a very simple difference in response processes can account for the empirical scale differences we found. Specifically, we assume that people who provide responses to a Likert scale are actually having subjective experiences at the granularity of the VAS, but are then forced to map their experience to the seven response categories of the Likert scale. We would like to stress that we think that the real difference in response processes will likely be much more complicated. However, it is interesting to explore to what extent such a simple thresholding process can already account for the empirical scale differences we found. To evaluate this, we map the empirical VAS measurements to a seven-point Likert scale using equally spaced thresholds (for details see Appendix G) and evaluate to what extent this thresholding explains observed differences between the Likert scale and the VAS.

As a first result, we examined to what extent the higher means that we found on the VAS in comparison to the Likert response scale could be explained by such thresholding. The detailed results of this analysis are presented in Appendix G. Across all items, the thresholding explained 28% (HDI $[12\%, 48\%]$) of the difference in means that we found initially between the two response scales. The thresholding process could also explain 70% (HDI $[23\%, 156\%]$) of the difference in mean item correlations, 35% (HDI $[-2\%, 83\%]$; note that the HDI includes zero) of the difference in the average magnitude of autocorrelations, and 52% (HDI $[24\%, 89\%]$) of that in skewnesses. In contrast, the thresholding process did not account for any of the differences that we found on external correlations, with all HDIs clearly including zero.

On means and skewnesses, the explanatory value of the thresholding process depended on the valence of the items. For both statistics, the thresholding process could only explain the effect of the response scale on negative valence items, with 76% (HDI $[38\%, 135\%]$) of the effect explained on item means, and 74% (HDI $[29\%, 157\%]$) on skewnesses, but not on positive valence items, with estimates very close to zero and symmetric HDIs around zero. For the mean item correlations and autocorrelations, the extent to which the thresholding was able to account for scale differences did not meaningfully depend on item valence, and for the external correlations, the HDIs were broad across both valences.

**Table 1:** Summary of scale differences in analyzed characteristics. NAs indicate that there were no zero-inflation parameters for the given outcome. Note that in the missing data model, apart from zero-inflation there was also one-inflation but no difference therein was visible between the response scales. “*No difference*” indicates that the respective 90% HDI included zero such that null effect in the population was not outruled.

Outcome	Mean	Variance	Zero-inflation
Mean	VAS 30% larger	VAS 31% larger	Likert 28% higher
Standard Deviation	No difference	No difference	Likert 27% higher
Skewness	Likert 57% larger	Likert 33% larger	NA
Multimodality	No difference	No difference	NA
RMSSD	No difference	Likert 6% larger	Likert 26% higher
AR(1)	VAS 30% larger	No difference	NA
Mean Abs Cor	VAS 13% larger	VAS 8% larger	NA
Correlation Structure	Very similar		NA
Criterion Correlations	VAS 44%-147% larger		NA
Missing Data	No difference	No difference	No difference
Duration	No difference	No difference	NA
Subjective Exp	No difference	No difference	NA

### Non-psychometric outcomes

#### Missing data

**Fig. 15:**
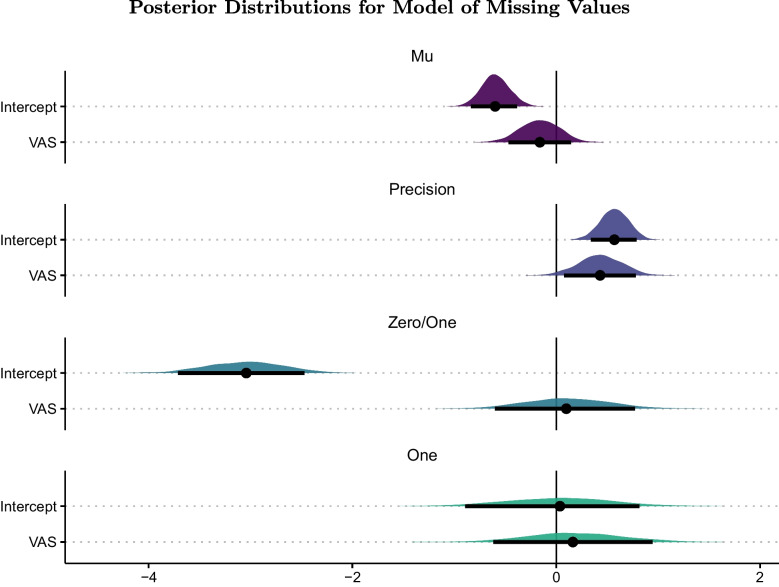
Posterior distributions of regression parameters in zero-inflated beta regression model for proportions of missing data. *Points* indicate medians of posteriors with 90% credible intervals defined by highest density intervals as *black lines* and distributions of the full posteriors. The intercept is defined as the average intercept across items with negative and positive valence in the Likert condition. *VAS* denotes the estimated slope of the VAS condition compared to the Likert condition. *Valence* indicates estimated difference between items with positive and negative valence. *VAS:valence* denotes the estimated interaction between the VAS condition and item valence. *Mean* denotes estimated effects on mean parameters of beta distribution, *Precision* estimated effects on inverse of variance, and *zero* estimated effects on the share of exact zero values. Means and zero parts are on logit scale (i.e., exponentiation yields odds, which divided by 1 + odds yield probability/proportion of zero values) and precision is on log scale (i.e., exponentiation leads to original scale)

We investigated group differences in the proportion of missing measurement points using a Bayesian zero-inflated beta regression model. The medians of the model-implied posteriors of the proportion of missing data were somewhat larger for Likert (0.36) than for VAS (0.33), but the HDI of the difference between the two overlapped with zero (HDI $[-0.10, 0.03]$). We therefore cannot conclude that the proportion of missing data is different across scales in the population (for details see Fig. 15 in Appendix B).

#### Duration

**Fig. 16:**
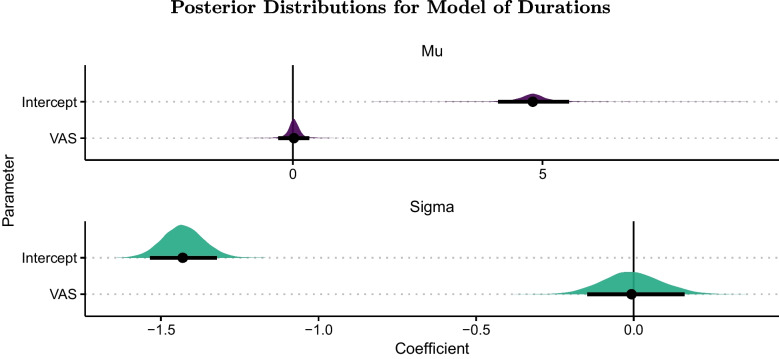
Posterior distributions of regression parameters in location-scale lognormal regression model for duration. *Points* indicate medians of posteriors with 90% credible intervals defined by highest density intervals as *black lines* and distributions of the full posteriors. The intercept is defined as the estimated duration in the Likert condition. *VAS* denotes the estimated slope of the VAS condition compared to the Likert condition. *Mu* denotes estimated effects on expectation parameter of lognormal distribution, *Sigma* estimated effects on standard deviation

We investigated scale differences in the time it took participants to fill out the EMA surveys, using a log-normal regression model. The medians of model-implied posteriors are similar for the two response scales (Likert: 135s; VAS: 137s), and the HDI of the difference overlaps with zero (median: 2s, HDI: [-7s, 10s]). We therefore conclude that the amount it takes to respond to the two scales is different. For the posteriors of all fixed effects, see Fig. 16 in Appendix B.

#### Post survey questionnaire

**Fig. 17:**
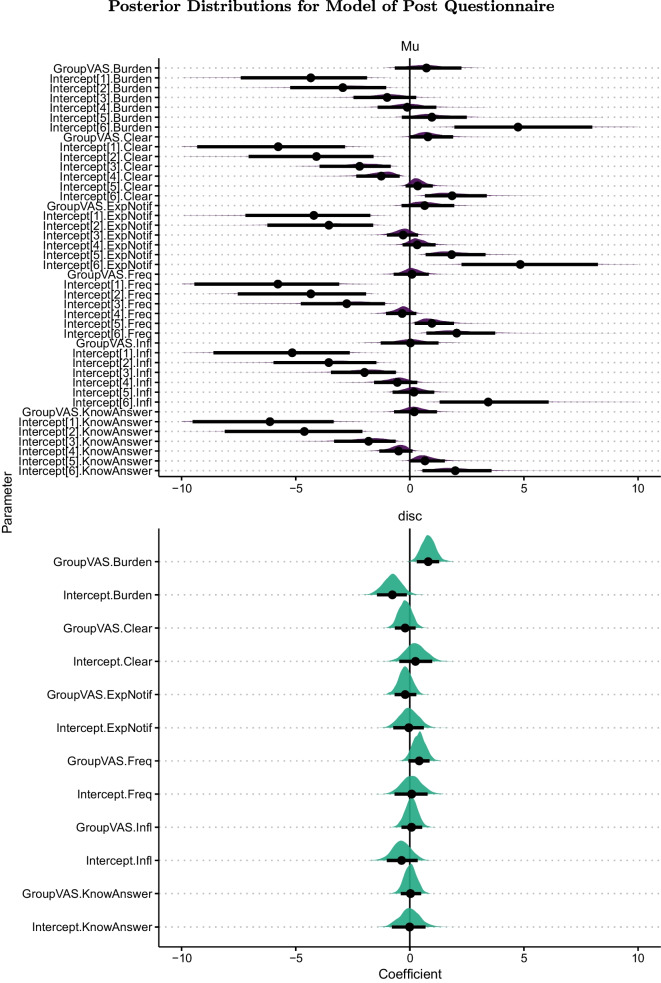
Posterior distributions of regression parameters in location-scale multivariate cumulative ordinal regression model for post questionnaire. *Points* indicate medians of posteriors with 90% credible intervals defined by the highest density intervals as *black lines* and distributions of the full posteriors. Intercepts of *Mu* parameters are estimated category thresholds on logit scale and group VAS parameters estimated slopes that are added to all thresholds in VAS condition compared to Likert condition. *disc* denotes latent discrimination parameter

**Fig. 20:**
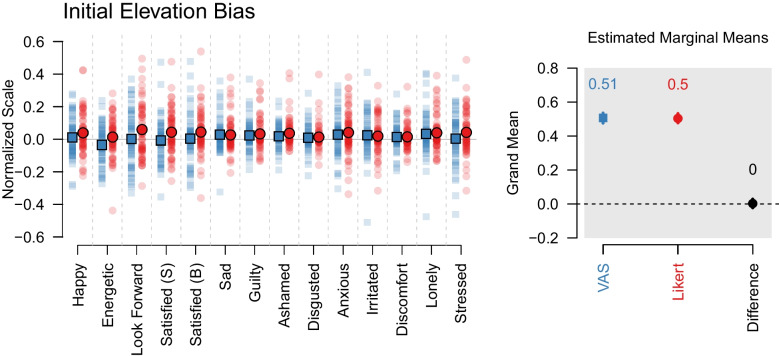
Data and summaries of the data (*left*) and posteriors of fixed effects of Bayesian multilevel model (*right*)

**Fig. 21:**
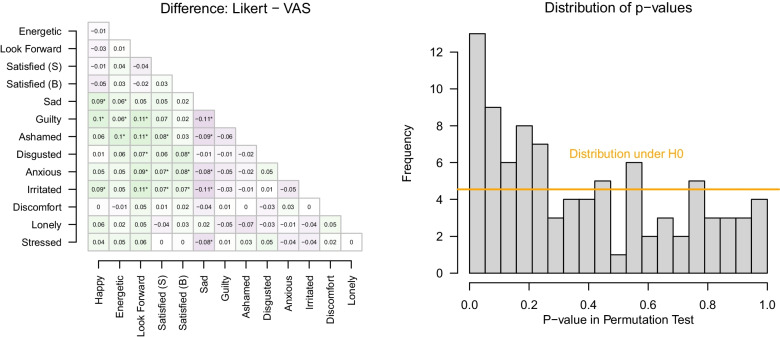
*Left*: Correlations in Likert group minus correlations in VAS group; *asterisks* indicate significance at the $\alpha =0.05$ level in a permutation test. *Right*: Distribution of *p* values in the permutation test

**Fig. 22:**
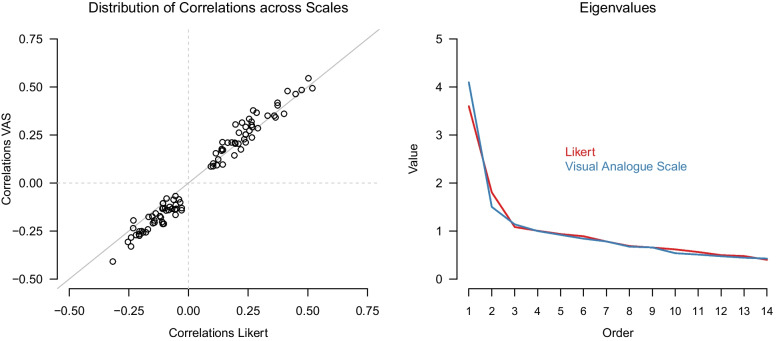
*Left*: Correlations in Likert group (*x*-axis) plotted against correlations in the VAS group (*y*-axis); *Right*: Eigenvalues of the correlation matrices in the Likert (*red*) and VAS (*blue*) groups

For 53 of the (28 Likert, 25 VAS) 119 participants we have responses to six questions reflecting on participants’ experience with the EMA survey. We performed inference on group differences with a multivariate cumulative ordinal regression and report the posteriors for all parameters in Fig. 17 in Appendix B. Here we report the medians and HDIs for the estimated marginal means of the difference (VAS minus Likert) between the scales. The difference is the difference in the answers to the questions about both scales, which are given on a seven-point Likert scale.

Participants rated responding to the VAS scale as moderately easier with the credible interval including 0 (median of difference: 0.350, HDI $[-0.143, 1.04]$. The perceived burden of the survey was higher in the VAS group but the credible interval did again overlap with zero (median of difference: 0.38, HDI $[-0.26, 1.31]$). The difficulty of knowing the answer was rated similar for the two scales (median of difference: 0.00, HDI $[-0.18, 0.18]$). The clarity was rated a bit higher for the Likert scale (median of difference: $-0.30$, HDI $[-0.81, 0.11]$. The frequency of the assessments was rated as being moderately higher in the VAS group, with the credible interval including 0 (median of difference: 0.350, HDI $[-0.14, 1.04]$). The rating of how difficult it was to know the answer to questions did not differ across scales (median=0.01, HDI $[-0.25, 0.22]$). The scales also did not differ visibly in how much participants felt that the survey influenced their lives (median = 0.00, HDI $[-0.64, 0.50]$). Overall, for all post survey questions, no or moderate descriptive effects could be found, and all credible intervals included zero, indicating that no reliable differences were present in the population.

### Summary of results

Table 1 summarizes the scale differences for all considered outcomes. For all outcomes, we performed inference on scale differences in the respective means. As described in the method and at the beginning of the results for the respective models, for some outcomes, we also modeled scale differences in the variances and the proportions of zeros or ones. Note that the table summarizes only the most important statistical estimates and inferences.

## Discussion

We reported the first experimental study comparing a Likert scale with a visual analog scale for measurements of affect in ecological momentary assessment. We found that means, mean absolute correlations, and lag-1 autocorrelations were reliably higher in the VAS group, skewness was more negative in the VAS group, and correlations with the external criteria – BSI and the DASS subscales – were considerably higher in the VAS group. We found no reliable differences between Likert and VAS groups regarding within-person standard deviations, RMSSD, initial elevation bias, the duration of measurements, and missing data. We also found that there was more zero inflation for means and standard deviations for the Likert scale, and that there was higher variance in means in the VAS group and in skewness in the Likert group.

We first discuss observed commonalities across scales and possible explanations for them, and then turn to the differences we found across scales and hypothesize what response processes brought them about. Based on these findings, we formulate preliminary recommendations for researchers using EMA and suggest future research to better understand the response processes triggered by different response scales.

### Commonalities across response scales

We inspected a number of qualitative patterns that we would generally expect in measures of momentary affect in a healthy sample and found all of them in both scales: We found higher means for positive valence items, positive skew for negative valence items and negative (positive) correlations of positive (negative) valence item means with the external criteria BSI/DASS. The descriptive plots shown in our paper also showed that the distributions of the characteristics were overlapping considerably for the two scales. We also found no reliable differences in missing data, the time it took participants to respond, and the ratings of six questions capturing the participant’s experience with the EMA study.

These similar patterns suggest that, in general, choosing one scale over the other will not lead to completely different results in terms of qualitative statistical patterns in relationships between the different items, their correlations with external criteria, or specific distributional characteristics of the measured affective dynamics. Assuming that our findings generalize to variations of the Likert and VAS scales and to other populations, this suggests for the EMA literature at large that major qualitative patterns do not depend on the response scale, which would imply that prior and future findings relating to these characteristics do not require to be replicated with a different response scale to ensure their generalizability across these response modalities.

These results about commonalities are generally in line with prior research comparing Likert and VAS in cross-sectional research. Specifically, prior research mostly found either no effects or effects of small magnitude on psychometric characteristics, but no major differences in qualitative results, such as in directions of correlations or differences in orders of magnitude (Lozano et al., 2008; Simms et al., 2019).

### Differences between response scales

Two scale differences stood out. The first is the finding that within-person means were higher in the VAS group. This likely also explains differences in skewness, which is in part driven by the mean since both scales are bounded. Statistically, the differences in means can be at least partially explained by two shifts in the distribution in the VAS group. First, the VAS condition led to overall higher responses when providing non-zero responses. In addition, in the VAS group we found $28\%$ fewer estimated exact zero values compared to the Likert scale and a substantial lower skewness. The finding of less zero values on the VAS is consistent with Voutilainen et al. (2016), who found reduced ceiling effects on VAS compared to a Likert scale. Although in our study, we found reduced floor effects but could not examine differences in ceiling effect, both studies indicate less extreme responses on the VAS. Van Laerhoven et al. (2004), however, found more extreme responses on the VAS in a sample of children, as well as Hasson and Arnetz (2005) in a sample of non-student non-pensioners. These findings suggest that respondents choosing a zero response on the Likert scale may at times select ratings on the VAS slightly above zero. This might indicate that the VAS captures variation in affect that occurs close to zero but is slightly above. Capturing this variation close to the scale end is especially relevant for items which are mostly scored close to the scale end, such as the negative valence items in our study.

The second major scale effect we identified was consistently higher correlations between baseline questionnaires and item means in the VAS group compared to the Likert group. We were surprised by the magnitude of these differences. The HDIs around the estimated marginal differences were very small, and their lower ends are far from zero, leading us to conclude that the means of items assessed with the VAS indeed are more strongly correlated with the four external criterion scales we considered. We visually inspected the scatter plots for each of the reported correlations with the criterion constructs and could not identify artifacts that could explain these results.

This result may in part be explained by the higher overall means and lower frequency of zero ratings on the VAS discussed above. Dynamics that are reflected in variation close to the scale minimum (or maximum) may only be captured by the VAS and these dynamics may give rise to between-person variation that covaries more strongly with the external indicators of general psychopathology than the remaining distribution that is captured by the Likert scale. However, replication as well as a proper understanding of this result are needed.

This finding ties into recent work on the value of mean affect and affect dynamics for predicting participant differences in mental health. Dejonckheere et al. (2019) found that affect dynamics offered little predictive information about constructs related to well-being and general psychopathology after controlling for mean levels. However, if our speculation about the importance of capturing state variation close to the scale extremes is correct, this would imply that a thoughtful choice of the response scale is required nonetheless because it affects the nature and amount of trait-relevant variation that is covered by the responses. Capturing such variation close to the extremes may shift the overall means and their correlations with external criteria. Thus, even if EMA research focuses on measuring stable trait levels instead of state variation and dynamics, choosing appropriate response scales is critical to avoid missing important state variation that shapes the overall mean. Adapting and extending recent statistical approaches that have been developed to capture effects of state variation on trait estimates and development might help testing whether our potential explanations and their implications regarding this result are valid (Neubauer et al., 2022).

Apart from those two key differences between Likert and VAS groups, we found that correlations between the same item at subsequent measurement points and correlations between different items at the same time point were larger in the VAS group. If these increased correlations are not due to systematic variation induced by a measurement process related to the VAS that is correlated across both measurement points and items, and unrelated to the construct of interest, these results suggest that the VAS scale captures the considered affective states more accurately.

We investigated whether scale differences could be accounted for by a simple thresholding process, in which we threshold the empirical VAS responses into a seven-point Likert scale. We found that such a process could account for a substantial part of the scale differences for within-person means, skewness, autocorrelations, and correlations at the same time point. For means and skewness, the thresholding process did so only for the negative valenced items. The thresholding process did not meaningfully account for any scale differences in external correlations. These results suggest that a thresholding process from a more granular experience to a less granular (Likert) scale may be part of an explanation for the observed scale differences. These findings about means and skewness are in line with our discussion above on how the VAS allows for higher means particularly on items producing more frequent values that are close to the scale minimum or maximum. However, since the thresholding process only explains some of the scale differences, this analysis also shows that more research is needed to better understand the response processes triggered by different response scales in EMA.

### Implications for researchers

Now, which scale is to be preferred for measurements in EMA studies? While choices about measurement instruments should never be made based on empirical results alone, and a detailed understanding of the measurement process is necessary to make definitive recommendations, our results allow us to make some preliminary recommendations.

The higher correlations between EMA item means and external criteria related to psychopathology with the VAS suggest that this scale captures more of a signal that we are interested in, especially in research relating to mental health. The smaller-scale effects of higher correlations between items at the same time point and higher correlations of the same item with itself at the previous time point in the VAS group lead to the same conclusion.

In addition, we saw that the means are higher for the VAS, concomitant with a lower frequency of exact zeros (i.e., the response scale minimum). These results indicate that the VAS may be able to better capture meaningful variation close to the scale end. This is especially relevant for items that generally tend to have low means, such as negative affect items in non-clinical populations. This variation may be lost in a Likert scale, since the corresponding responses are all equal to zero. The higher means may also be part of the explanation for the higher criterion correlations. Therefore, based on our findings, we would tentatively recommend the use of the VAS when assessing subjective affective states or similar constructs, or other constructs that may yield informative variance close to the scale limits. One could argue that the variation of the VAS scale around scale ends is not meaningful, because respondents are simply unable to select a precise number due to fundamental physical/haptic limitations. However, in a recent study (Cloos et al., n.d.) showed that adults are able to provide very precise responses on a VAS scale when asked to respond with a specific number.

### Limitations & suggestions for future research

We were not able to vary all possible design choices and compared only a specific Likert scale against a specific VAS scale, for the specific population of university students, using a specific (between-person) experimental design. Therefore, additional research is needed to complement and assess the generalizability of our results. Here, we suggest a number of directions in which our study could be extended.

Some of our findings were rather surprising and we therefore would welcome a relatively direct replication of our results to see how generalizable they are. It would also be interesting to know whether the results are different with the same materials, but with a different population. Regarding the latter, our sample consisted mostly of women, so it would be interesting to see in a more balanced sample whether there are any gender differences. It would also be interesting to what extent our results generalize to children, non-student adults, or people diagnosed with certain mental health problems.

Studies employing more fine-grained variations of the response scale, such as vertical and horizontal response scales, different numbers of response categories with or without labels on the Likert scale, and VAS implementations that vary the presence and location of the initial slider position might be explored in randomized metastudy designs to get thorough insight into the psychometric effects of these variations on response scale differences (Baribault et al., 2018). Since our literature review of prior studies comparing response scales showed that results tend to be inconsistent across different study characteristics, varying more than one factor at a time within metastudy designs might be an important design approach to be able to disentangle interactions between multiple factors.

Our between-person experiment did not allow us to compare how the responses on the Likert and VAS scale compare for an individual person. A within-person experiment, presenting both response scales to each person for each item, would allow us to directly estimate the mappings between Likert and VAS scales and how persons differ in this mapping. However, within-person experiments in this context come with the downside that sequential effects are very likely: Answering an item on a Likert (or VAS) scale with a given response would almost certainly influence the subsequent response on the VAS (Likert) scale. Despite this limitation, within-person experiments may provide useful evidence complementary to our between-person experiment. A within-person experiment could also directly test the question of whether variation close to the scale ends in the VAS is meaningful. This could be done by subsetting those intervals of VAS measurement during which the corresponding Likert measurement is constant. If the VAS measurements are systematically correlating with other variables in those intervals, we would conclude that the variation is systematic.

There are several ways in which one could better investigate the extent to which persons find it easy or burdensome to provide answers on a given scale. One could ask more specific questions in a survey after (or during) the EMA period asking about the experience of persons filling in the scale. We asked about the burden associated with providing responses and how difficult it was to know the answer. But one could ask much more detailed questions or provide open text responses to learn about a person’s experience. In this context, it would also be interesting to compare the extent of careless responding across scales. We visually inspected the time series of all persons, identifying only a single person (VAS group) with clear careless responding, which did not allow us to draw any strong conclusions about careless responding. However, larger samples may allow us to draw such conclusions. In such a context, careless responding could also be modeled formally using exploratory latent Markov factor analysis (e.g., Vogelsmeier et al., 2024)

Another promising avenue for further research is directly interrogating the response processes using qualitative methods such as interviewing participants to better understand the measurement process, by revealing how participants understand and use the response scale, and how the scales might differ in this regard (Beatty and Willis, 2007; Desimone and Floch, 2004; Ryan et al., 2012; Wolf et al., 2021). Insights from interviews, combined with quantitative psychometric studies, could provide the basis for developing theories that characterize the processes that unfold during measurement in EMA studies and the role of the response scale in these processes. Such theories could be translated into psychometric models for response processes such as multinomial processing trees (Erdfelder et al., 2009) and item response trees (Jeon and De Boeck, 2016), which can be adapted to items with Likert or VAS response scales (Klauer et al., 2014; Jeon and De Boeck, 2016).

Apart from these process models, a general measurement theory that is adapted to the particularities of intensive longitudinal data would help to translate insights from interviews and further sources into more detailed cognitive process models (Castro-Alvarez et al., 2022; Crayen et al., 2017). Available measurement models that distinguish between trait, state, and dynamic effects could be particularly informative when multi-item scales are used in EMA designs to implement psychometric models that link the responses with underlying constructs (Castro-Alvarez et al., 2022; Crayen et al., 2017). Of course, using multiple item scales affects the number of constructs that can be assessed within resource constraints and without risking increased measurement reactivity (Kaurin et al., 2023). In combination with multi-item scales, the response format could also be varied experimentally for external criterion measures (e.g., psychopathology trait measures) to fully disentangle common method variation caused by the response format from actual correlated construct variance (Eid et al., 2023).

### Conclusion

Our study provided the first experimental comparison between Likert and VAS response scales for measurements in EMA studies. While the measurements were overall quite similar, we identified a number of reliable differences across scales, which tentatively suggest the use of the VAS for items measuring affective experiences in EMA. Our findings contribute to a growing body of quantitative, qualitative, and theoretical research assessing which response scale is best in which situation in EMA studies.

## Figures and Tables

**Fig. 6:**
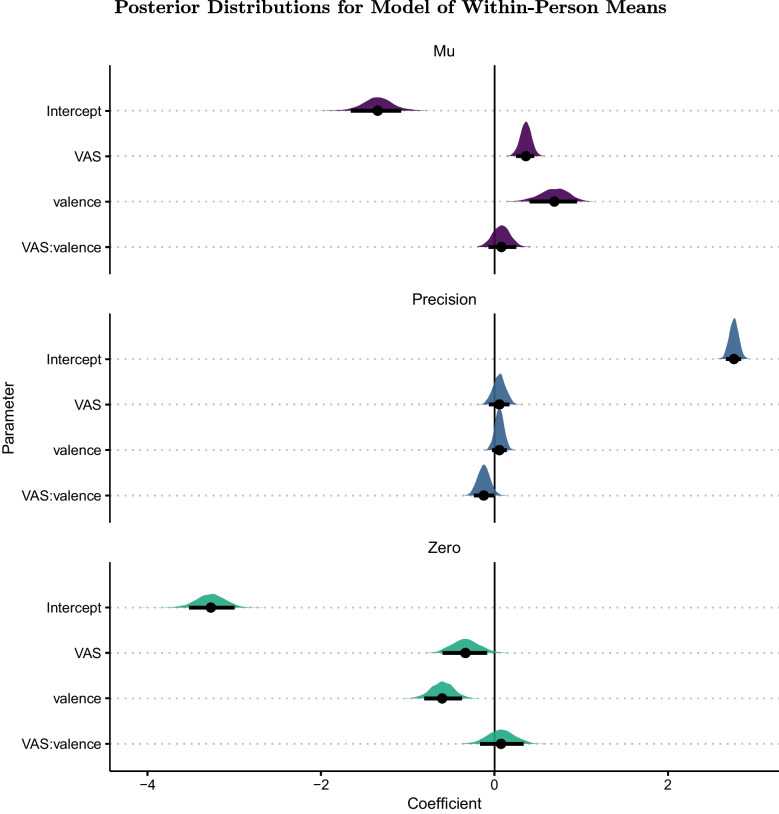
Posterior distributions of regression parameters in zero-inflated multilevel beta regression model of within-person means. *Points* indicate medians of posteriors with 90% credible intervals defined by highest density intervals as *black lines* and distributions of the full posteriors. The intercept is defined as the average intercept across items with negative and positive valence in the Likert condition. *VAS* denotes estimated slope in comparison to Likert condition. *Valence* indicates the estimated difference between items with positive and negative valence. *VAS:valence* denotes the estimated interaction between the VAS condition and item valence. *Mu* denotes estimated effects on *Mu* parameters of beta distribution, *Precision* estimated effects on inverse of variance, and *zero* estimated effects on the share of exact zero values. *Mu* and zero parts are on logit scale (i.e., exponentiation yields odds, which divided by 1 + odds yield probability/proportion of zero values) and precision is on log scale (i.e., exponentiation leads to original scale)

**Fig. 7:**
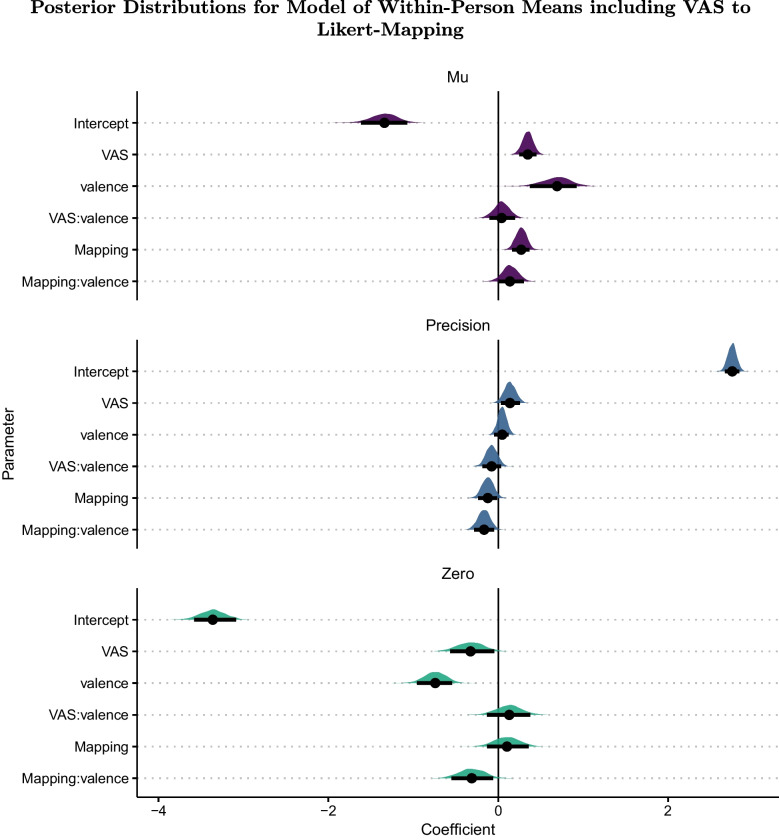
Posterior distributions of regression parameters in zero-inflated multilevel beta regression model of within-person means, including the VAS mapped to Likert approach. *Points* indicate medians of posteriors with 90% credible intervals defined by highest density intervals as *black lines* and distributions of the full posteriors. The intercept is defined as the average intercept across items with negative and positive valence in the Likert condition. *VAS* denotes the estimated slope of the VAS condition compared to the Likert condition. *Valence* indicates estimated difference between items with positive and negative valence. *VAS:valence* denotes the estimated interaction between the VAS condition and item valence. *Mean* denotes estimated effects on mean parameters of beta distribution, *Precision* estimated effects on inverse of variance, and *zero* estimated effects on the share of exact zero values. Means and zero parts are on logit scale (i.e., exponentiation yields odds, which divided by 1 + odds yield probability/proportion of zero values) and precision is on log scale (i.e., exponentiation leads to original scale)

**Fig. 8:**
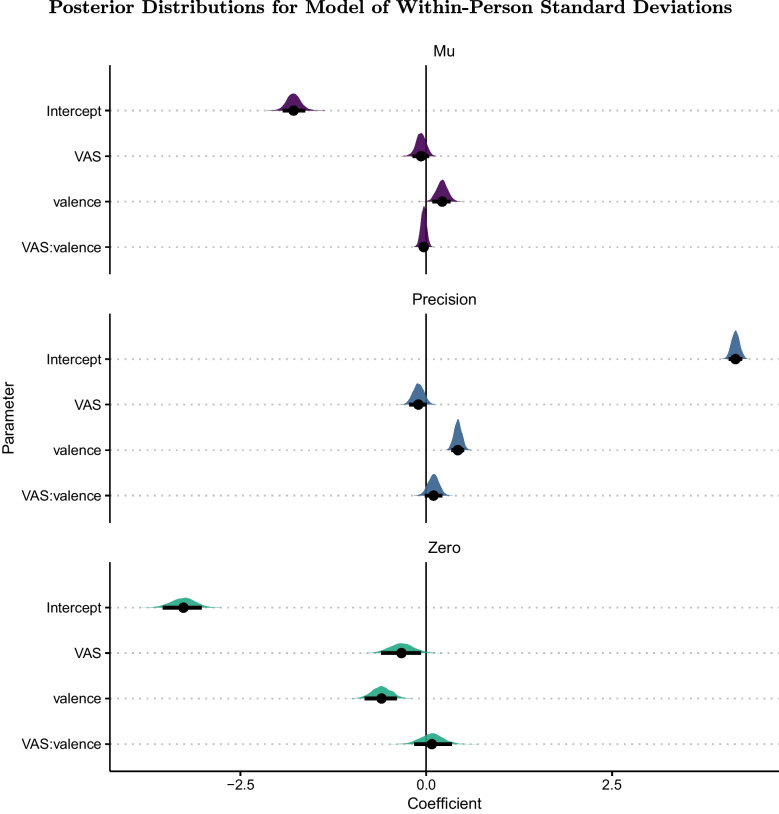
Posterior distributions of regression parameters in zero-inflated multilevel beta regression model of within-person standard deviations. *Points* indicate medians of posteriors with 90% credible intervals defined by highest density intervals as *black lines* and distributions of the full posteriors. The intercept is defined as the average intercept across items with negative and positive valence in the Likert condition. *VAS* denotes the estimated slope of the VAS condition compared to the Likert condition. *Valence* indicates estimated difference between items with positive and negative valence. *VAS:valence* denotes the estimated interaction between the VAS condition and item valence. *Mu* denotes estimated effects on *mu* parameters of beta distribution, *Precision* estimated effects on inverse of variance, and *zero* estimated effects on the share of exact zero values. *Mu* and zero parts are on logit scale (i.e., exponentiation yields odds, which divided by 1 + odds yield probability/proportion of zero values) and precision is on log scale (i.e., exponentiation leads to original scale)

**Fig. 9:**
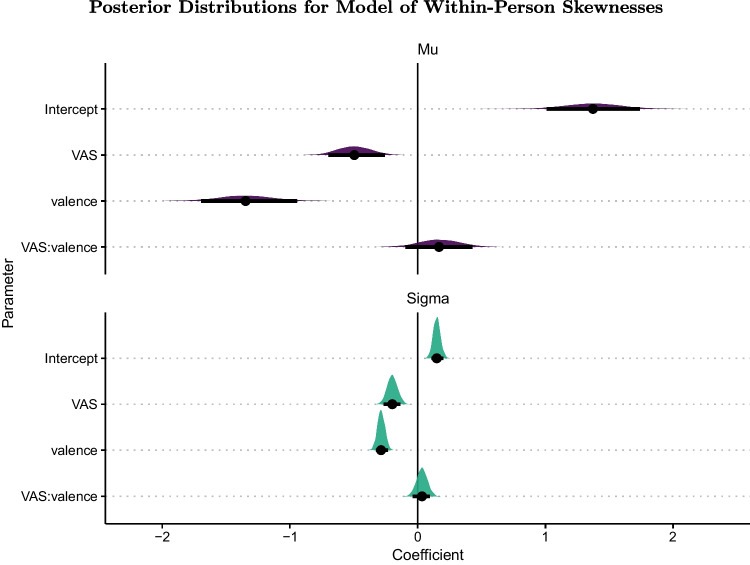
Posterior distributions of regression parameters in location-scale multilevel regression model of within-person mean skewnesses. *Points* indicate medians of posteriors with 90% credible intervals defined by highest density intervals as *black lines* and distributions of the full posteriors. The intercept is defined as the average intercept across items with negative and positive valence in the Likert condition. *VAS* denotes the estimated slope of the VAS condition compared to the Likert condition. *Valence* indicates estimated difference between items with positive and negative valence. *VAS:valence* denotes the estimated interaction between the VAS condition and item valence. Variance parameters are on log scale (i.e., exponentiation leads to original scale)

**Fig. 10:**
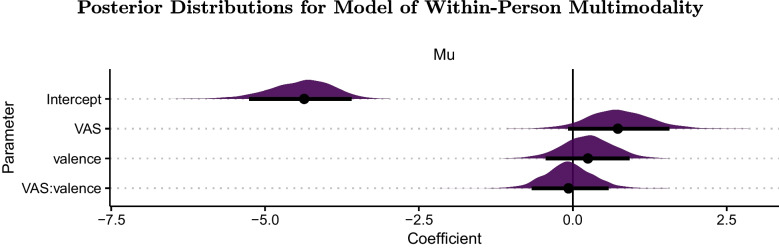
Posterior distributions of regression parameters in location-scale multilevel regression model of within-person multimodality. *Points* indicate medians of posteriors with 90% credible intervals defined by highest density intervals as *black lines* and distributions of the full posteriors. The intercept is defined as the average intercept across items with negative and positive valence in the Likert condition. *VAS* denotes the estimated slope of the VAS condition compared to the Likert condition. *Valence* indicates estimated difference between items with positive and negative valence. *VAS:valence* denotes the estimated interaction between the VAS condition and item valence. Parameters denote estimated log-odds (i.e., exponentiation yields odds, which divided by 1 + odds yield probability/proportion of multimodality)

**Fig. 11:**
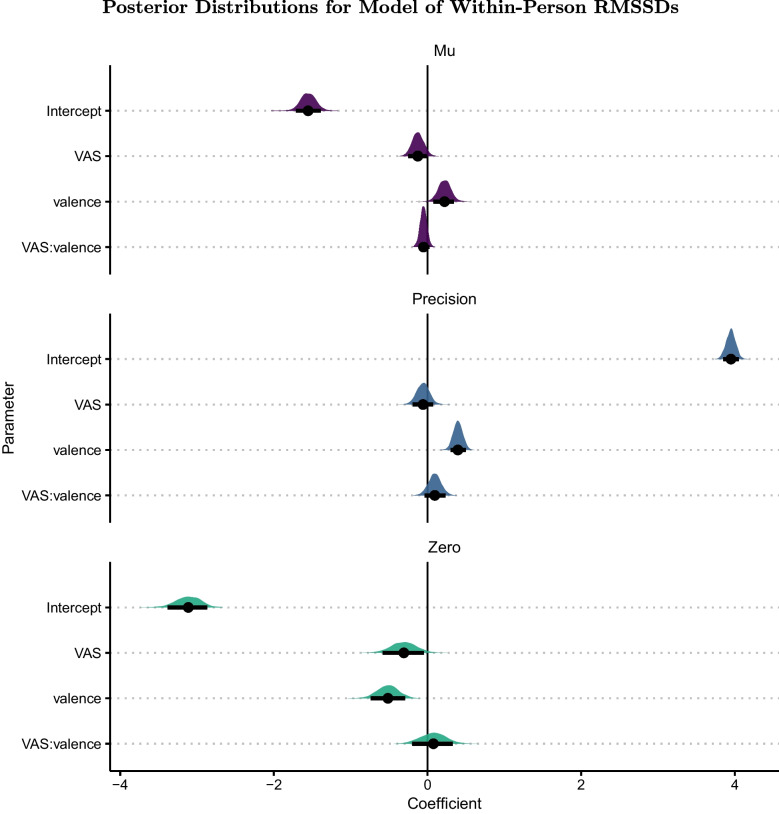
Posterior distributions of regression parameters in zero-inflated multilevel beta regression model of within-person RMSSDs. *Points* indicate medians of posteriors with 90% credible intervals defined by highest density intervals as *black lines* and distributions of the full posteriors. The intercept is defined as the average intercept across items with negative and positive valence in the Likert condition. *VAS* denotes the estimated slope of the VAS condition compared to the Likert condition. *Valence* indicates estimated difference between items with positive and negative valence. *VAS:valence* denotes the estimated interaction between the VAS condition and item valence. Means and zero parts are on logit scale (i.e., exponentiation yields odds, which divided by 1 + odds yield probability/proportion of zero values) and precision is on log scale (i.e., exponentiation leads to original scale)

**Fig. 12:**
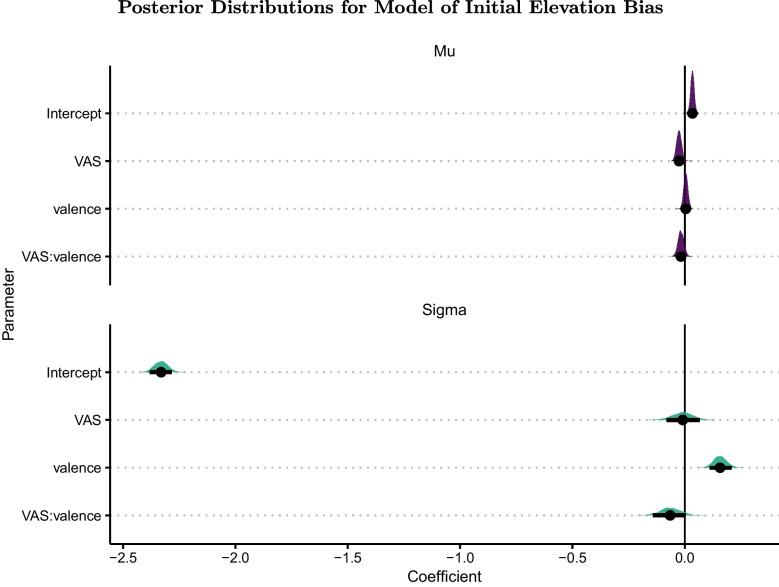
Posterior distributions of regression parameters in location-scale multilevel regression model of initial elevation bias. *Points* indicate medians of posteriors with 90% credible intervals defined by highest density intervals as *black lines* and distributions of the full posteriors. The intercept is defined as the average intercept across items with negative and positive valence in the Likert condition. *VAS* denotes the estimated slope of the VAS condition compared to the Likert condition. *Valence* indicates estimated difference between items with positive and negative valence. *VAS:valence* denotes the estimated interaction between the VAS condition and item valence. Variance is on log scale (i.e., exponentiation leads to original scale)

**Fig. 13:**
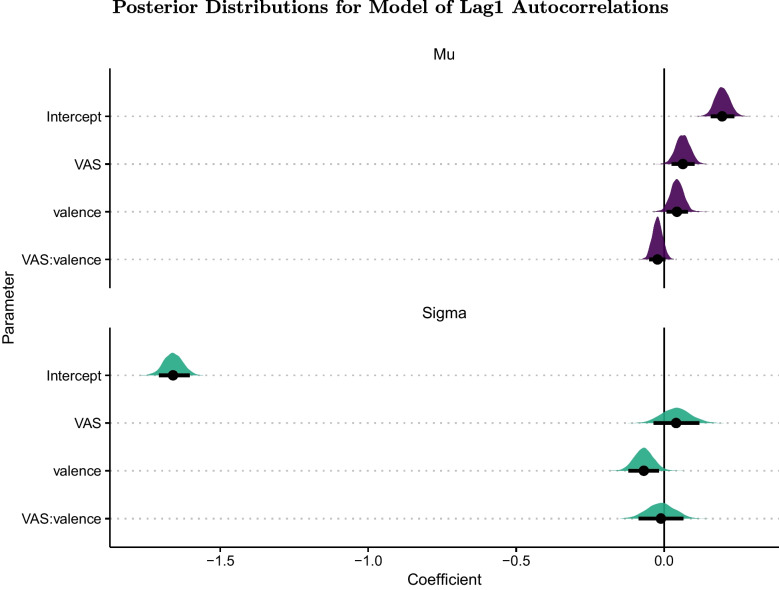
Posterior distributions of regression parameters in location-scale multilevel regression model of within-person lag1 autocorrelations. *Points* indicate medians of posteriors with 90% credible intervals defined by highest density intervals as *black lines* and distributions of the full posteriors. The intercept is defined as the average intercept across items with negative and positive valence in the Likert condition. *VAS* denotes the estimated slope of the VAS condition compared to the Likert condition. *Valence* indicates estimated difference between items with positive and negative valence. *VAS:valence* denotes the estimated interaction between the VAS condition and item valence. Variance is on log scale (i.e., exponentiation leads to original scale)

**Fig. 14:**
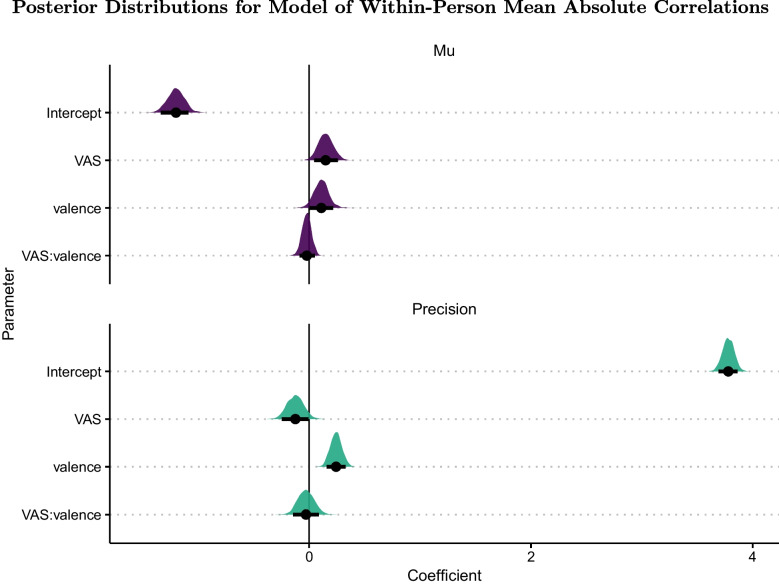
Posterior distributions of regression parameters in multilevel location-scale beta regression model of within-person mean absolute correlations. *Points* indicate medians of posteriors with 90% credible intervals defined by highest density intervals as *black lines* and distributions of the full posteriors. The intercept is defined as the average intercept across items with negative and positive valence in the Likert condition. *VAS* denotes the estimated slope of the VAS condition compared to the Likert condition. *Valence* indicates estimated difference between items with positive and negative valence. *VAS:valence* denotes the estimated interaction between the VAS condition and item valence. *Mu* denotes estimated effects on *mu* parameters of beta distribution, *Precision* estimated effects on inverse of variance. *Mu* are on logit scale (i.e., exponentiation yields odds, which divided by 1 + odds yield probability/proportion of zero values) and precision is on log scale (i.e., exponentiation leads to original scale)

**Fig. 18:**
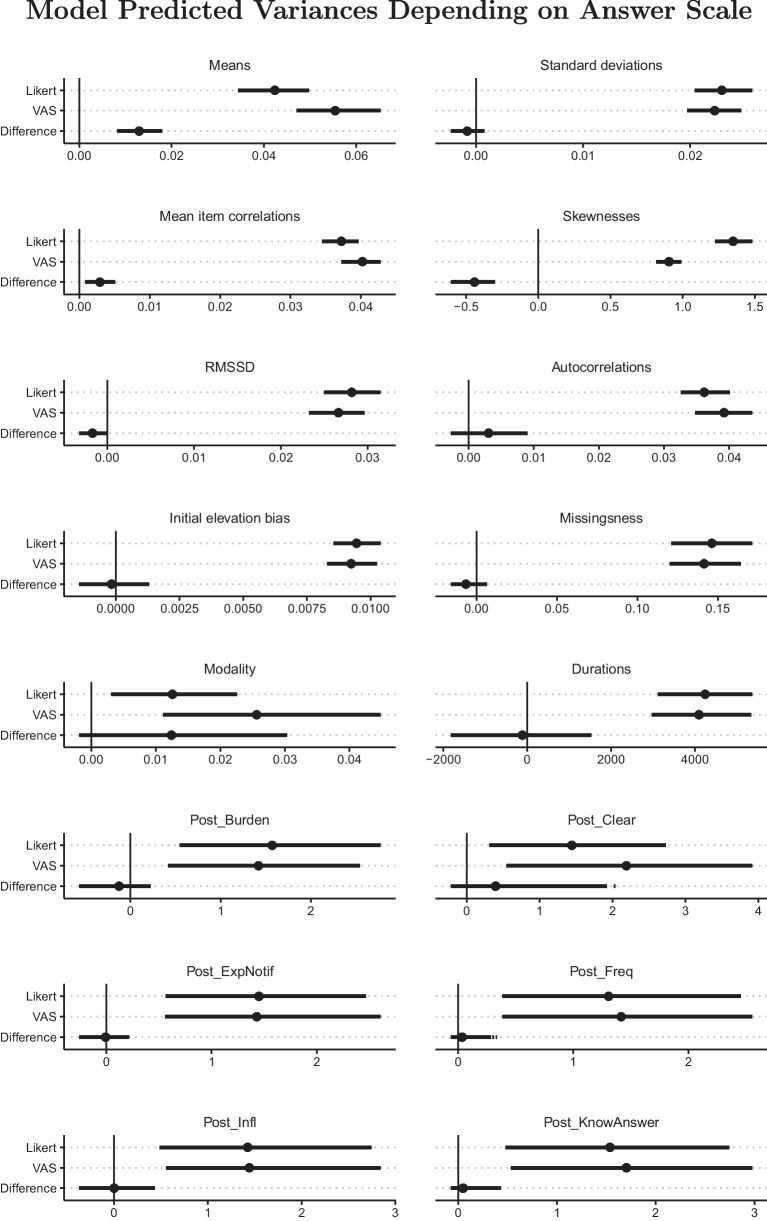
Estimated marginal variances and differences therein between Likert and VAS conditions in the different Bayesian regression models. Positive estimated difference-parameters indicate larger variance on VAS and negative differences indicate larger variance on Likert scale

**Fig. 19:**
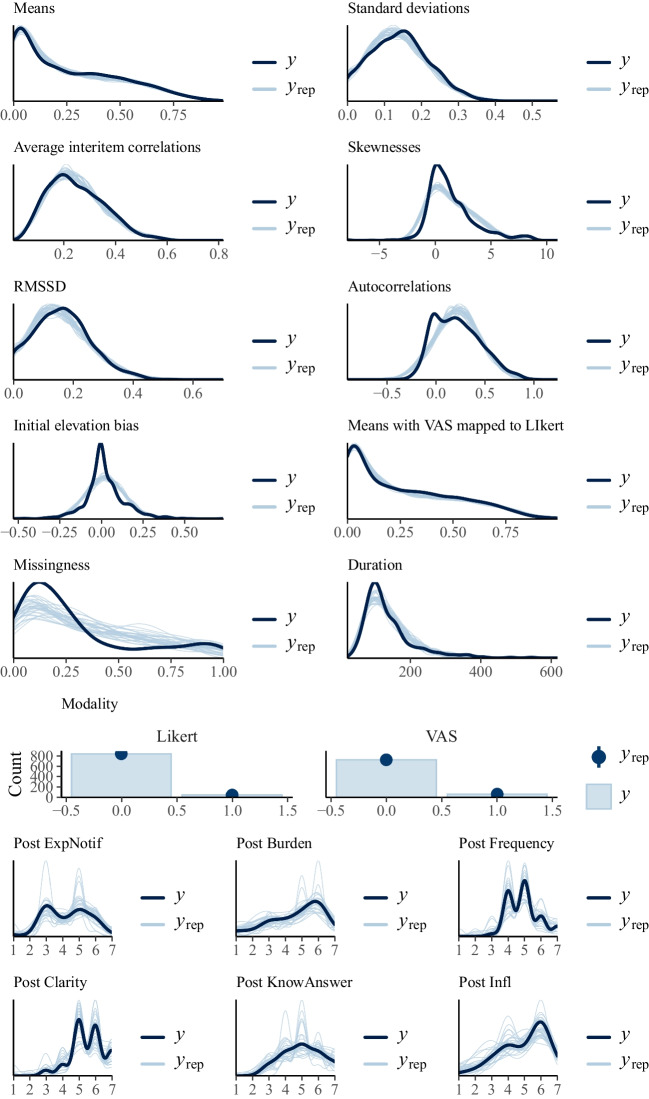
Posterior predictive checks comparing empirical distributions ($\textit{y}$) with 30 posterior draws ($\textit{y}_{rep}$) from fitted Bayesian multilevel models

**Fig. 23:**
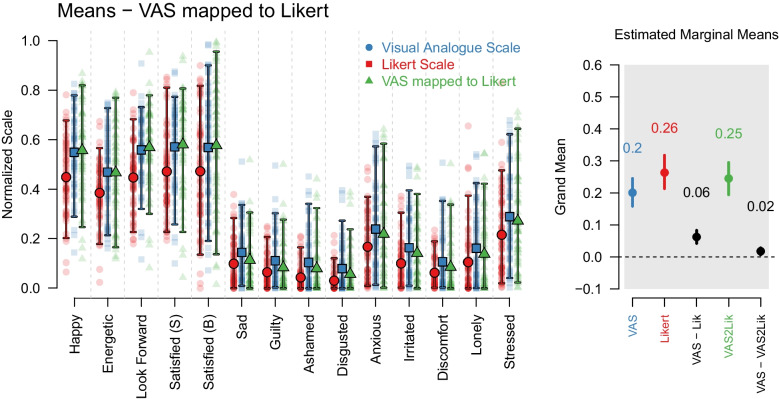
Same results as in panel **A** of Fig. 2 but with a third response scale, which is constructed by thresholding the VAS into a seven-point Likert scale

**Fig. 24:**
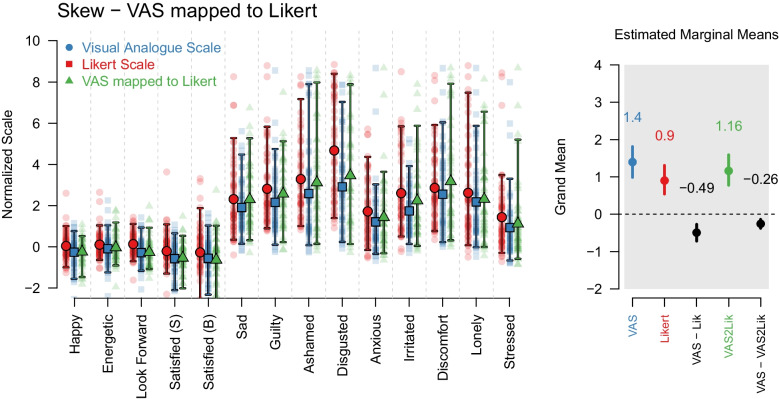
Same results as in panel **C** of Fig. 2 but with a third response scale, which is constructed by thresholding the VAS into a seven-point Likert scale

**Fig. 25:**
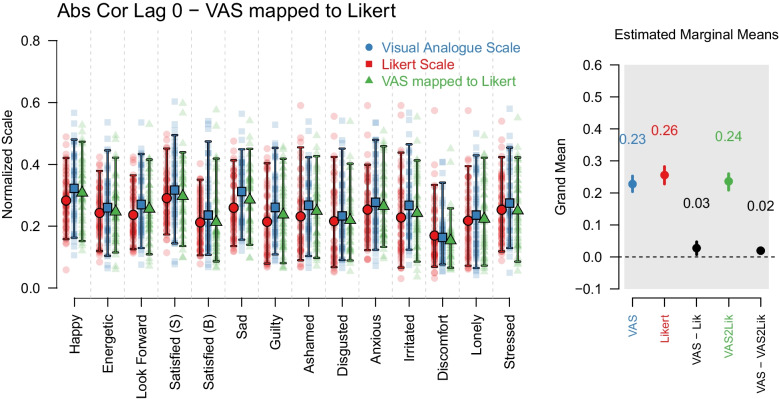
Same results as in panel **A** of Fig. 4 but with a third response scale, which is constructed by thresholding the VAS into a seven-point Likert scale

**Fig. 26:**
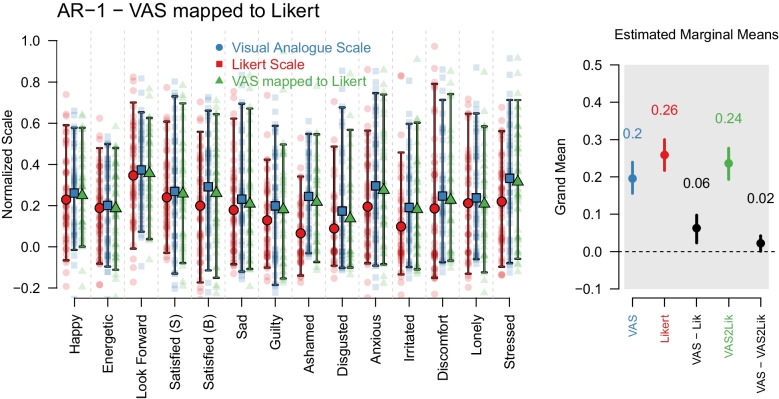
Same results as in panel **A** of Fig. 4 but with a third response scale, which is constructed by thresholding the VAS into a seven-point Likert scale

**Fig. 27:**
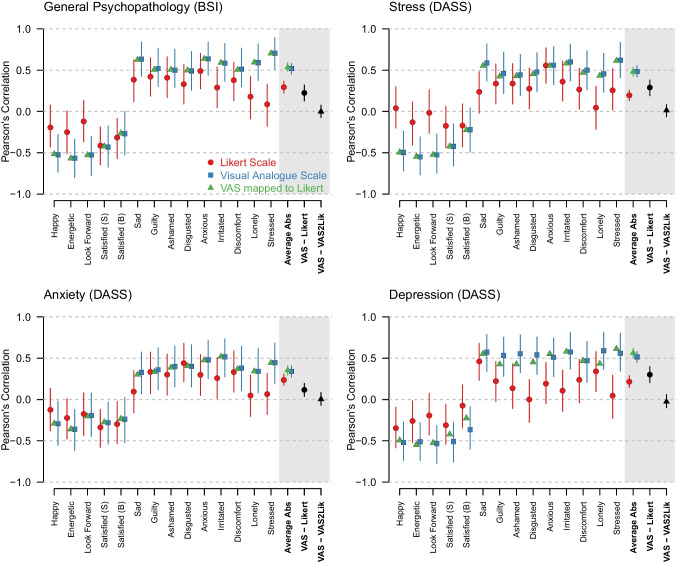
The same results as in Fig. 5 but with the correlations added between the respective external validation variable and measures constructed by thresholding the VAS into a seven-point Likert scale as above

## Data Availability

All results in the paper are based on within-person aggregates computed from multivariate time series. We share these aggregate measures at https://github.com/jmbh/LikertvsVASPaper, which allows the reader to reproduce all descriptive and modeling results we report in the main text and the appendices. Due to the data sharing policy of the NSMD consortium, which collected the data, we are not allowed to share the raw multiple multivariate time series data without restrictions. However, the raw data can be shared upon reasonable request for the purpose of replicating our findings and for new data analysis projects by contacting Anne Roefs (https://www.maastrichtuniversity.nl/).

## A Detailed questions on experience with EMA survey

The questionnaire participants filled out after the EMA survey included six questions about their experience with the EMA survey, which are potentially related to the used response scale:

1. *How did you experience filling out all notification surveys?* and the response scale was a seven-point Likert scale with the categories (1) *It was extremely difficult*, (2) *It was very difficult*, (3) *It was slightly difficult*, (4) *It was neither easy nor difficult*, (5) *It was fairly easy*, (6) *It was very easy*, and (7) It was extremely easy.
2. *How burdensome was answering the notification surveys?* with answer categories (1) *Extremely burdensome*, (2) *Very burdensome*, (3) *Burdensome*, (4) *Quite burdensome*, (5) *Fairly burdensome*, (6) *Slightly burdensome*, and (7) *Not burdensome at all*.
3. *How was the frequency of the surveys (i.e. the number of times you were asked to fill in a survey every day)?* with answer categories (1) *Extremely low*, (2) *Low*, (3) *A bit low*, (4) *Fine*, (5) *A bit high*, (6) *High*, and (7) *Extremely high*.
4. *Were the questions clear?* with answer categories (1) *Extremely unclear*, (2) *Very unclear*, (3) *Unclear*, (4) *Neither clear nor unclear*, (5) *Clear*, (6) *Very clear*, and (7) *Extremely clear*.
5. *How difficult was it for you to know the answers to the questions?*, with answer categories (1) *Extremely difficult*, (2) *Very difficult*, (3) *Difficult*, (4) *Neither easy nor difficult*, (5) *Easy*, (6) *Very easy*, and (7) Extremely easy.
6. *How influential was your participation in this study on your day-to-day life?* with answer categories (1) *Extremely influential*, (2) *Very influential*, (3) *Influential*, (4) *Quite influential*, (5) *Fairly influential*, (6) *Slightly influential*, and (7) *Not influential at all*.

## D Posterior predictive checks

For posterior predictive checks, 30 draws were created from the posterior predictive distributions of all models. The results, presented in Fig. 19 indicate that for most models, the appropriate overall fit was achieved, as indicated by large overlap between the distributions of the posterior predictive draws indicated by blue lines, and the empirical distributions indicated by black lines. For three models, fit appeared worse than for the others. Specifically, for the model of skewnesses, the model appears to predict larger variation around the lower part of the distribution (values below five), but less outliers in the upper part between values of five and 10. This leads to the lower part of the posterior predictive distribution being more leptokurtic for the lower values than the empirical distribution, and the upper part being flatter. We implemented a skewed Gaussian distribution as an alternative but this only mildly improved fit at the cost of worse interpretability. As the current model does not appear to be bad and the estimated marginals were well in accordance with descriptive statistics, this is why we chose to remain with the current model. Second, for autocorrelations, the empirical distribution appears moderately bimodal, whereas the posterior predictive distribution is unimodal and misses the lower empirical mode which is centered at about zero. We were unsure about the statistical and theoretical nature of the second, missed mode. Since overall, the fit appeared ok, we decided to remain with the current model. For the initial elevation bias, the empirical distribution appears to have smaller kurtosis (i.e., more platykurtic) than the posterior predictive distribution. The estimated marginals, however, were well in accordance with the empirical descriptive statistics, why we decided to remain with the well-interpretable Gaussian model. Finally, for missingness, similar to autocorrelations, the posterior predictive misses a second mode, which can be seen in the upper values (close to 100% missing values) in the empirical distribution. The model-estimated marginals across the two response scales aligned well with the empirical descriptive statistics, and the fit appeared adequate across most of the distribution, leading to the decision to retain the current model. Overall, all posterior predictive checks indicated moderately or well-fitting models. To further enhance fit, future research could explore alternative modeling approaches, such as mixture distributions, to better capture the moderate multimodality observed in some distributions.

## E Results for initial elevation bias

Here we are looking into initial elevation bias (IEB), which describes the phenomenon that in experience sampling and daily diary studies participants often start with higher values in the first few measurement occasions than in the rest of the time interval (Anvari et al., 2023; Shrout et al., 2018; Cerino et al., 2022; Arslan et al., 2019). We define the IEB by taking the difference between the mean over the observations on the first eight time points minus the mean over the observations on the remaining 76 time points. Choosing a cutoff earlier or later in the time series leads to similar results. Figure 20 shows the results for the IEB. Both the data and the multilevel model suggest that there is no IEB in any of the two groups, and therefore there is also no scale effect.

## F Additional results on scale differences in within-person correlation matrices

The left panel of Fig. 21 shows the difference between the correlations in the Likert group and the correlations in the VAS group (Likert minus VAS). We see mostly negative difference values for correlations between items with different valences, and mostly positive differences for items with the same valences. Since the former tend to be negative and the latter positive, this means that correlations overall tend to be larger in absolute value in the VAS group. This is in line with the aggregate scale effect tested in the multilevel model shown in Fig. 4 in the main text.

We performed inference on the group difference in the population for each correlation using a permutation test. The asterisk next to the difference values in the left panel of Fig. 21 indicates significance with a threshold of $\alpha = 0.10$. From this test, we would conclude that 22 correlations are different in the population. This amounts to $24.2 \%$ of all correlations, compared to the false-positive rate of $10\%$, we would expect with $\alpha = 0.05$. However, given the consistent signs in the differences, we would expect higher absolute correlations in the VAS group for almost all item pairs, but these differences were too small to be detected with our permutation test for a lack of power.

The above results are dependent on the specific choice of *alpha*. To get a better aggregate view of whether the correlations are the same across the two groups, we inspect the distribution of *p* values in the right panel of Fig. 21. We see that *p* values are skewed towards 0, indicating that the null hypothesis is rejected for a sizeable number of correlations. The orange horizontal line indicates the uniform distribution we would expect if all correlations are equal across groups in the population.

To infer group differences in each correlation within the population, we conducted a permutation test with the null hypothesis that the correlations in the Likert and VAS groups are equivalent. The *p* values are shown in the left panel of Fig. 21. If we apply a significance threshold of $\alpha = 0.05$ we obtain significant group differences for 12 correlations, which corresponds to 13.18% of all tests, which is larger than the expected false-positive rate of 5% with $\alpha = 0.05$.

Next to comparing the correlation between a given pair of items between the Likert and VAS groups, it is also interesting to investigate how different the overall structure of the two mean correlation matrices is. The left panel of Fig. 22 shows the correlations in Likert group (*x*-axis) plotted against correlations in the VAS group (*y*-axis). In line with Fig. 21 we see that correlations in the VAS group are systematically larger in absolute value. However, we also see that the correlations are highly correlated across scales (Pearson’s correlation = 0.986). This means that, while the correlations seem to be overall higher in the VAS group, the correlations that are negative (positive) in the Likert group are also negative (positive) in the VAS group, and the correlations that are small (large) in the Likert group are also small (large) in the VAS group.

Finally, we considered the eigendecomposition to get a summary of the structure of the correlation matrices. The right panel of Fig. 22 shows the eigenvalues for both groups. We see that the first eigenvalue of the VAS group is a bit higher, which makes sense since there is a strong factor structure in the data and the overall correlations in the VAS group are larger. However, we again see that the eigenvalue distribution is highly similar across groups, which again shows that while the correlations seem to be consistently higher in the VAS group, the remaining structure in the correlation matrices seems to be very similar.

## G Effect of thresholding VAS into seven-Point Likert

One possible explanation for differences between the VAS and Likert scales could be that participants query a value on a continuous dimension that is well captured by VAS but then threshold these continuous values into the seven Likert categories. While it is likely that reality is more complicated, we are interested to what extent such a simple thresholding process explains differences between Likert and VAS.

Many thresholding rules are possible, but here we choose the perhaps most obvious one in the absence of any additional information, which is eight equally spaced thresholds on the [0, 100] interval of the VAS, which allow us to threshold the VAS measurements into the seven categories of the Likert scale. Such thresholding can, in principle, explain differences between the scales whenever the VAS measurements are not uniformly distributed, which they typically are not.

Here we show the results for all characteristics for which we found a reliable scale difference (see Table 1) and add the results of the scale obtained by mapping the empirical VAS data to a seven-point Likert scale with the above-mentioned procedure:

Figure 27 shows the same results as Fig. 5 but added the correlations between the VAS thresholded into a Likert scale and the four external validation scales. We see that the thresholding does not explain the differences between Likert and VASs.

## H Demographics of participants in both groups

Table 2 presents the mean and standard deviation of participants’ age, along with frequencies for gender, continent, and history of mental disorder diagnosis separately for each group.

**Table 2:** Demographics and frequencies separated by group

Variables	Likert	VAS
Age	$\mu = 22.3$ ($\sigma = 4.36$)	$\mu = 21.5$ ($\sigma = 3.78$)
Gender: Male	8	8
Gender: Female	55	45
Gender: Other	0	3
Continents: EU	42	52
Continents: Asia	15	3
Continents: Other	6	1
Diagnosis: Yes	12	13
Diagnosis: No	51	43

## I Means and standard deviations of responders vs. non-responders

Table 3 shows the mean and standard deviation of the studied variables separately for each group.

**Table 3:** Means and standard deviations on studied variables between non-responders and responders

Variables	Non-responders	Responders
Happy	0.56 (0.26)	0.50 (0.16)
Energetic	0.44 (0.23)	0.42 (0.15)
Look Forward	0.60 (0.23)	0.50 (0.15)
Satisfied (S)	0.51 (0.27)	0.52 (0.18)
Satisfied (B)	0.49 (0.31)	0.52 (0.23)
Sad	0.16 (0.15)	0.12 (0.11)
Guilty	0.17 (0.17)	0.09 (0.09)
Ashamed	0.16 (0.19)	0.07 (0.09)
Disgusted	0.09 (0.16)	0.05 (0.08)
Anxious	0.30 (0.17)	0.20 (0.16)
Irritated	0.16 (0.14)	0.13 (0.12)
Discomfort	0.13 (0.18)	0.08 (0.10)
Lonely	0.14 (0.19)	0.13 (0.14)
Stressed	0.32 (0.21)	0.25 (0.18)

## Footnotes

1 https://aspredicted.org/ej6jp.pdf

## Code availability

We share all analysis scripts, which include all preprocessing scripts and which allow the reader to reproduce all analyses and figures shown in the paper from the summary statistics (within-person means, etc), which we also share at https://github.com/jmbh/LikertvsVASPaper.
